# Inhibition of β-catenin dependent WNT signalling upregulates the transcriptional repressor *NR0B1* and downregulates markers of an A9 phenotype in human embryonic stem cell-derived dopaminergic neurons: Implications for Parkinson’s disease

**DOI:** 10.1371/journal.pone.0261730

**Published:** 2021-12-23

**Authors:** John M. Haynes, Shanti M. Sibuea, Alita A. Aguiar, Fangwei Li, Joan K. Ho, Colin W. Pouton

**Affiliations:** 1 Stem Cell Biology Group, Monash Institute of Pharmaceutical Sciences, Monash University, Parkville, Victoria, Australia; 2 Badan Pengawas Obat dan Makanan, Jakarta, Indonesia; Rutgers University, UNITED STATES

## Abstract

In this study we investigate how β-catenin-dependent WNT signalling impacts midbrain dopaminergic neuron (mDA) specification. mDA cultures at day 65 of differentiation responded to 25 days of the tankyrase inhibitor XAV969 (XAV, 100nM) with reduced expression of markers of an A9 mDA phenotype (*KCNJ6*, *ALDH1A1* and *TH*) but increased expression of the transcriptional repressors *NR0B1* and *NR0B2*. Overexpression of *NR0B1* and or *NR0B2* promoted a loss of A9 dopaminergic neuron phenotype markers (*KCNJ6*, *ALDH1A1* and *TH*). Overexpression of *NR0B1*, but not *NR0B2* promoted a reduction in expression of the β-catenin-dependent WNT signalling pathway activator *RSPO2*. Analysis of Parkinson’s disease (PD) transcriptomic databases shows a profound PD-associated elevation of *NR0B1* as well as reduced transcript for *RSPO2*. We conclude that reduced β-catenin-dependent WNT signalling impacts dopaminergic neuron identity, *in vitro*, through increased expression of the transcriptional repressor, *NR0B1*. We also speculate that dopaminergic neuron regulatory mechanisms may be perturbed in PD and that this may have an impact upon both existing nigral neurons and also neural progenitors transplanted as PD therapy.

## Introduction

Parkinson’s disease is a debilitating disease affecting 1–2% of individuals over 50 years of age [1]. Patients with Parkinson’s disease suffer a variety of well-documented motor and non-motor symptoms. The motor symptoms include hypokinesia, muscle rigidity and shaking tremor [2], while the non-motor symptoms include depression, apathy, anxiety, hallucinations, sleep disorders, urinary urgency, nocturia, sexual dysfunction, dysphagia, fecal incontinence and paresthesia [3]. The classically described motor symptoms of Parkinson’s disease result from the loss of tyrosine hydroxylase (TH) positive dopaminergic (A9) neurons of the substantia nigra pars compacta. This results in a deficit of striatal dopamine and impaired motor function. Current pharmacotherapies encourage production of, or mimic or modulate the post-synaptic effects of dopamine. Newer therapies target increased survival of remaining neurons [4], while large scale trials of dopaminergic neural progenitor therapy are planned or underway (E.g. European Stem-PD, NYSTEM-PD, CiRA trial).

We know a great deal about the derivation of dopaminergic neurons from our understanding of mouse development. As the embryo develops, midbrain dopaminergic neuron development is largely under the control of sonic hedgehog (Shh) and β-catenin-dependent Wnt signalling [5–7]. After patterning, β-catenin-independent Wnt signalling suppresses β-catenin-dependent Wnt signalling to facilitate differentiation and maturation events [8]. Human pluripotent stem cell differentiation protocols attempt to mimic this sequence of events such that, maturing mouse and also human dopaminergic neural progenitors show early expression of transcription factors such as EN1/2, OTX2, FOXA2, LMX1A, MSX1, NEUROG2 and late stage factors such as ASCL1, NR4A2, PITX3 [9–13]. At maturation, PITX3 expression is important in defining mature midbrain dopaminergic neurons since it is highly localized to the midbrain [14], particularly the ventral substantia nigra [15]. Although PITX3 is highly localized to the midbrain, the rate limiting enzyme responsible for the synthesis of dopamine, tyrosine hydroxylase (TH), is commonly used as the definitive marker of dopaminergic phenotype. At maturity, substantia nigra (A9) dopaminergic neurons can be identified by expression of KCNJ6 (G Protein-Activated Inward Rectifier Potassium Channel 2 [16]), aldehyde dehydrogenase (ALDH1A1, [17]) and SOX6 (SRY, Sex Determining Region Y-Box 6, [18]). Ventral tegmental area (A10) dopaminergic neurons are often identified with calcium binding protein (calbindin, CALB1) and / or OTX2 [16, 18]. Significantly, the WNT signalling inhibitor dickkopf-3 has been linked to both the maintenance of *Lmx1a* and *Pitx3* expression in mice, and also to elements of subtype (A9 v A10) specification [19]. The role of WNTs in regulating biological process is also expanding beyond developmental programming, thus WNT signalling may have a role to play in some genetic forms of PD [20] which is consistent with the idea that WNT signalling is involved in neuron survival [21, 22].

Transplantation therapies for PD favour differentiating dopaminergic neuronal progenitors (~ day 20) as they offer improved connectivity and integration with host neurons as they mature (for reviews see [23, 24]). To that end there has been a continual and iterative push to create better dopaminergic neuron differentiation paradigms and/or purify dopaminergic neuronal progenitors (for example, see [25, 26]). In contrast with our understanding of early dopaminergic progenitor development, we have little understanding of what supports dopaminergic phenotype later in development, or indeed, after transplantation into humans. In this study, we cultivate pluripotent stem cell derived neurons for 65 days *in vitro*, and assess their transcriptomic profiles following a 25-day (day 40–65) incubation with either the canonical WNT signalling mimetic CHIR99025 (CHIR) or the canonical WNT signalling inhibitor, XAV969 (XAV). We subsequently overexpress three genes of interest (*NEUROD1*, *NR0B1* and *NR0B2*) and assess their impact upon dopaminergic phenotype. Our data indicate a complex WNT regulatory environment in maturing cultures and a profound regulation of dopaminergic neuronal phenotype, particularly by XAV. Analysis of PD transcriptomic databases indicates changes in endogenous WNT signalling accompanying the loss of neurons as well as markers of a dopaminergic A9 neuronal phenotype. These data lead us to suggest that, even after patterning, and at a time point beyond that at which they would be transplanted, maturing dopaminergic neurons are still strongly influenced by WNT signalling. This has major implications for the transplantation of neural progenitors for PD where the maintenance of dopaminergic phenotype during integration and beyond maturation may still be subject to influence by endogenous brain chemistry.

## Materials and methods

Parkinson’s disease array databases: For this analysis we accessed 11 array database sets to compare substantia nigra from PD and age-matched control tissues. GEO accession numbers: GSE20163 [27], GSE20141 [27]; GSE20164 [28]; GSE20333 (Edna et al. [Unpublished]); GSE7307 [29]; GSE7621 [28]; GDS3128 [30]; GDS3129 [30]; GSE54282 [31]; GSE43490 [32]; GSE8397 [33]. These datasets used any of five Affymetrix (GPL201, GL570, GPL17047, GPL97 and GPL96) or Agilent (GPL6480) platforms. S1 Table includes the specific data sets used for this study. For each gene of interest, transcript expression was standardized to 12 housekeeper genes (TRFC, RPLP0, PGK1, PPIA, ACTN1, B2M, GUSB, HPRT1, STAT1, ACTB, GAPDH). Given the wide range of platforms used, we kept analysis to a simple paired t-test design where each set of PD and control values from a single database contributed to an n of 1. Where lateral vs medial nigral tissue was used (for example [33]), we have used the average of both lateral and nigral expression to maintain consistency with whole nigral studies.

### Human ESC culture

The cell line used in this study expresses eGFP under the control of LMX1A (as described previously [34]). Briefly, the reporter hESC line (H9^LMX1A/eGFP^) was cultured, feeder free, on recombinant human laminin 521 (1μg cm^-2^, Life Technologies, Australia) using Essential 8 medium (Life Technologies, Australia). For hESC differentiation the method of [35] was employed. Briefly, on Day 0 of differentiation, hESCs were seeded onto Matrigel coated plates at a density of 120,000 cells cm^-2^, in 100% Midbrain Differentiation Media 1 (MDM1: Knock-out Serum Replacement containing: 15%, 1% Glutamax, 1% Non-Essential Amino Acids, 1% Penicillin/Streptomycin, 0.1% β-Mercaptoethanol, 100nM LDN-193189 and 10μM SB431542). On Day 1, media was replaced with MDM2: MDM1 containing sonic hedgehog (100ng/mL) and purmorphamine (2μM). On Day 3, media was replaced with MDM3: MDM2 containing CHIR99026 (CHIR, 3μM). After eight days (Day 11) the media was gradually replaced with maturation media (Neurobasal Medium containing 1% Penicillin/Streptomycin, 1% Glutamax, 2% B27 Supplement without Retinoic Acid as well as 20ng/mL each of BDNF and GDNF, 200μM Ascorbic Acid, 2.5μM DAPT, 1ng/mL TGFβ-3, 0.5mM dibutyryl cyclic AMP). The maturation media was completely replaced every two days. From days 40 to 65 of differentiation CHIR (1μM) or XAV939 (XAV, 100nM), or vehicle, were added to cultures. On the day of cells were harvested for quantitative reverse transcriptase PCR (qPCR) or immunocytochemistry.

### Overexpression of *NEUROD1 / NR0B1 / NR0B2*

Cultures were differentiated as described above and matured until day 55. At this time, cultures were transfected with NEUROD1 (SC118625, Origene), NR0B1 (SC319624, Origene), NR0B2 (SC122920, Origene) or blank plasmids (PCMV6XL5, Origene) according to manufacturer’s instructions. At days 1, 2, 6, 7, 9,10 post-transfection, NEUROD1 transcript and protein levels were established using qPCR and immunolabelling (we used days 3, 6 and 9 for NR0B1/2).

### qPCR

Quantitative PCR was undertaken using a method previously described [36]. RNA was extracted from 10^6^ cells using ISOLATE II Micro Kit (Bioline) according to manufacturer’s instruction. Briefly, cell lysis buffer is added and cell lysate was briefly centrifuged at 1000x*g* for five seconds, followed by homogenization with 70% (v/v) ethanol. Homogenized lysate was transferred to a small column with silica membrane and centrifuged to allow RNA binding to the membrane. After the wash steps, the RNA was eluted with 15 μL RNase-free water. Total RNA concentration was measured via the NanoDrop Spectrophotometer (ND-1000, ThermoFisher Scientific). Agarose gel electrophoresis was used to confirm the absence of DNA. To convert RNA to complementary DNA (cDNA), a SensiFast cDNA Synthesis Kit (Bioline) was used. Briefly, the extracted RNA was transferred to a Polymerase Chain Reaction (PCR) tube with 1 μL Reverse Transcriptase and 4 μL TransAmp Buffer which was then brought up to 20 μL with RNase-free water. Tubes were placed in a thermal cycler (Applied Biosystem) set to the protocol stipulated in the Bioline manual. Converted cDNA samples were stored at -20°C until further analysis. Three technical replicate reactions were performed on samples aggregated from at least three independently differentiating wells using the Bioline Sensifast SYBR No-ROX One Step Kit according to the manufacturer’s specifications. Relative quantification of gene expression was obtained using the ratio Ct values of target genes to mean Ct values to housekeepers, TBP1 & HPRT1.The list of Taqman probes is shown in S2 Table while S3 Table shows delta Ct values for each replicate.

### Immunocytochemistry and immunohistochemistry

Human brain sections from PD (three females aged 74, 65 and 64) and control subjects (three females aged 76, 67 and 64) were fixed to gelatin subbed microscope slides and fixed with 4% (w/v) paraformaldehyde (Merck, Australia) in PBS. Day 65 cultured neurons were fixed with 4% (w/v) paraformaldehyde (Merck, Australia) in PBS. Preparation for imaging was identical for both human tissues cultured cells. Samples were permeabilized with 0.05% (v/v) Triton™ X-100 (PBST; Sigma-Aldrich) in PBS for 20 minutes at room temperature and then blocked with 3% (v/v) normal donkey serum (Merck, Australia) in PBS for 20 minutes then incubated overnight in PBST with primary antibodies at 4°C (Abcam Australia, S4 Table). Samples were then incubated with secondary antibodies (Abcam Australia, S4 Table) diluted in PBST for 2 hours at room temperature prior to counterstaining with Hoechst 33342 (1:5000; Thermo Fisher Scientific). Fluorescence images were captured using a Nikon Ti A1R inverted confocal microscope (Nikon Instruments, Japan). Donor tissues were received from the Victorian Brain Bank: Project number 17.22. Histopathological examination of tissues confirmed diagnosis of PD in PD, but not control specimens. Monash University Human research ethics committee granted exemption from requiring ethics approval since deidentified human tissue sections were obtained from the Victorian brain bank network.

### Putative transcription factor binding sites

To better understand the differential regulation of genes by WNT signalling modulators and NEUROD1 we used MotifMap [37], to identify potential gene regulatory motifs an arbitrary 10,000 base pairs up and 10,000 base pairs downstream of transcription start sites. Later, we used two other web-based tools to assess agreement between different platforms in defining putative transcription factor binding sites: ConTra V3 [38] and LASAGNA [39]. Broadly speaking, the results across the three platforms were largely compatible although the LASAGNA and MotifMap showed more consistency in identify and quantifying numbers of putative transcription factor binding sites.

### Statistical analyses

For most qPCR we used repeated measures one-way ANOVA with post-hoc Dunnett’s test to identify differences in Δ^ct^ values. Where qPCR showed undetectable expression (ie. >40 cycles) the non-parametric Kruskal-Wallis test with post-hoc Dunn’s test was used (where undetectable values were ranked as equal lowest expression). For expression in graphs, data sets are shown as log_2_ Δ^ct^ or log_2_ ΔΔ^ct^. For NEUROD1 and NR0B1/2 overexpression studies Student’s paired t-tests, or repeated measures two-way ANOVA with post-hoc Dunnett’s test were used where times after transfection (not including t = 0) were arranged in columns and analysis identified main column effect (ie. differences in expression over the time course of the study). All statistical analyses were performed with GraphPad Prism 8.

## Results

### Expression profiles of developing cultures change over time

**Fig 1:**
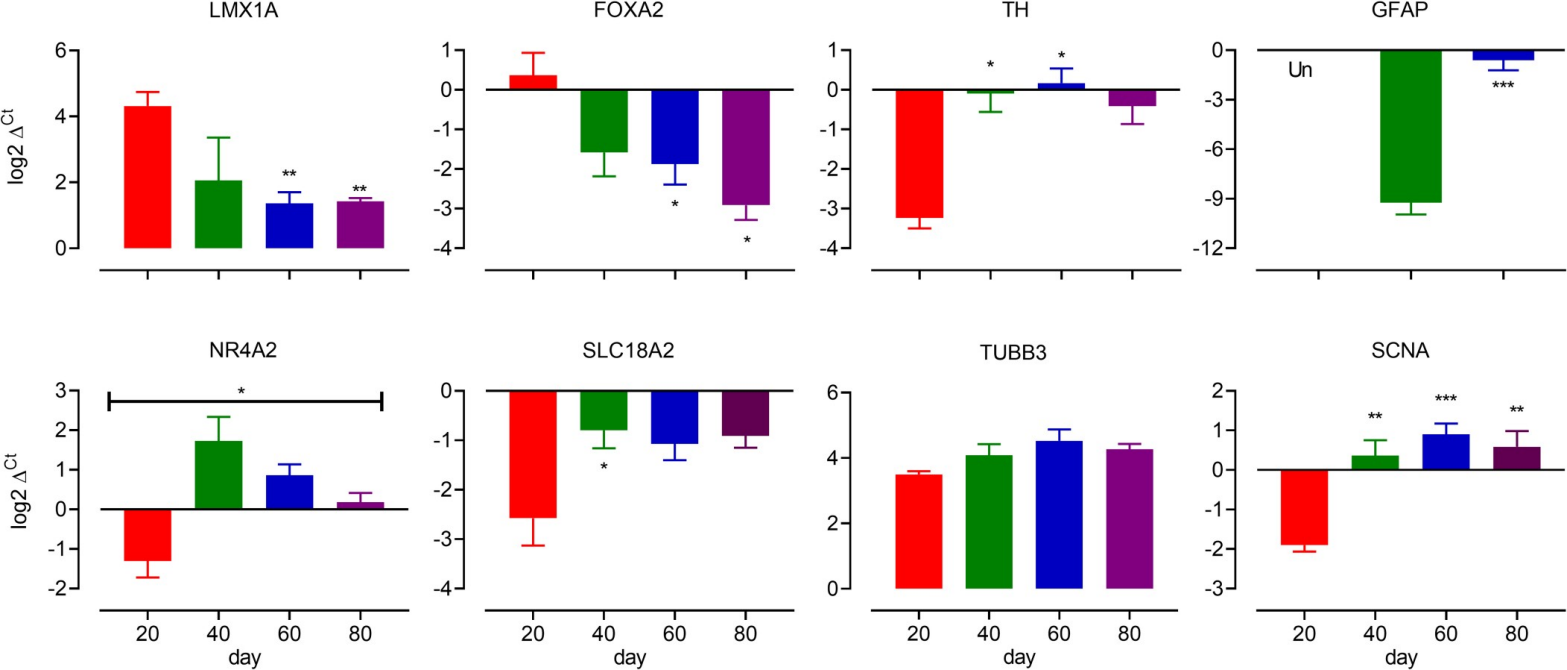
Changes in expression of a number of dopaminergic neuronal markers during differentiation. Comparison of dopaminergic gene markers gene expression at days 20,40, 60 and 80 of differentiation. Data are expressed as log_2_ Δ^Ct^ values for presentation. Most statistical analysis used One-way ANOVA followed by Dunnett’s multiple comparison test, day 20 was used for comparison, (n = 3–26). *, ** and *** indicate P<0.05, <0.01 and <0.001, respectively. Since GFAP was not detected at day 20 (Un, >40 cycles) and not measured at day 80, we used a Student’s t-test to compare values at day 40 and 60.

We used qPCR to track the fate of dopaminergic neuronal cultures from day 20 to day 80 of differentiation. There were significant decreases in LIM Homeobox Transcription Factor 1α (*LMX1A*) and Forkhead Box A2 (*FOXA2*) over time, although *LMX1A* expression appeared to plateau from day 40. There were also elevations of α-synuclein (*SCNA*), Solute Carrier Family 18 Member A2 (*SLC18A2* or *VMAT2*) and tyrosine hydroxylase (*TH*) (Fig 1). These results are consistent with the idea that maturing cultures reduce expression of early markers, such as *FOXA2* and *LMX1A* while increasing expression of markers associated with a more mature dopaminergic neuronal phenotype, *TH*, *SLC18A2* and *SCNA*. The expression of Nuclear Receptor Subfamily 4 Group A Member 2 (*NR4A2*), a transcription factor associated with dopaminergic neurogenesis and maintenance, peaked at day 40 before slowly reducing over time. Developing cultures also showed a relatively constant expression of β3-tubulin (*TUBB3*), but an elevation of glial associated fibrillary acid (*GFAP*) transcript over time, indicating that astrocyte like cells develop after neurons.

**Fig 2:**
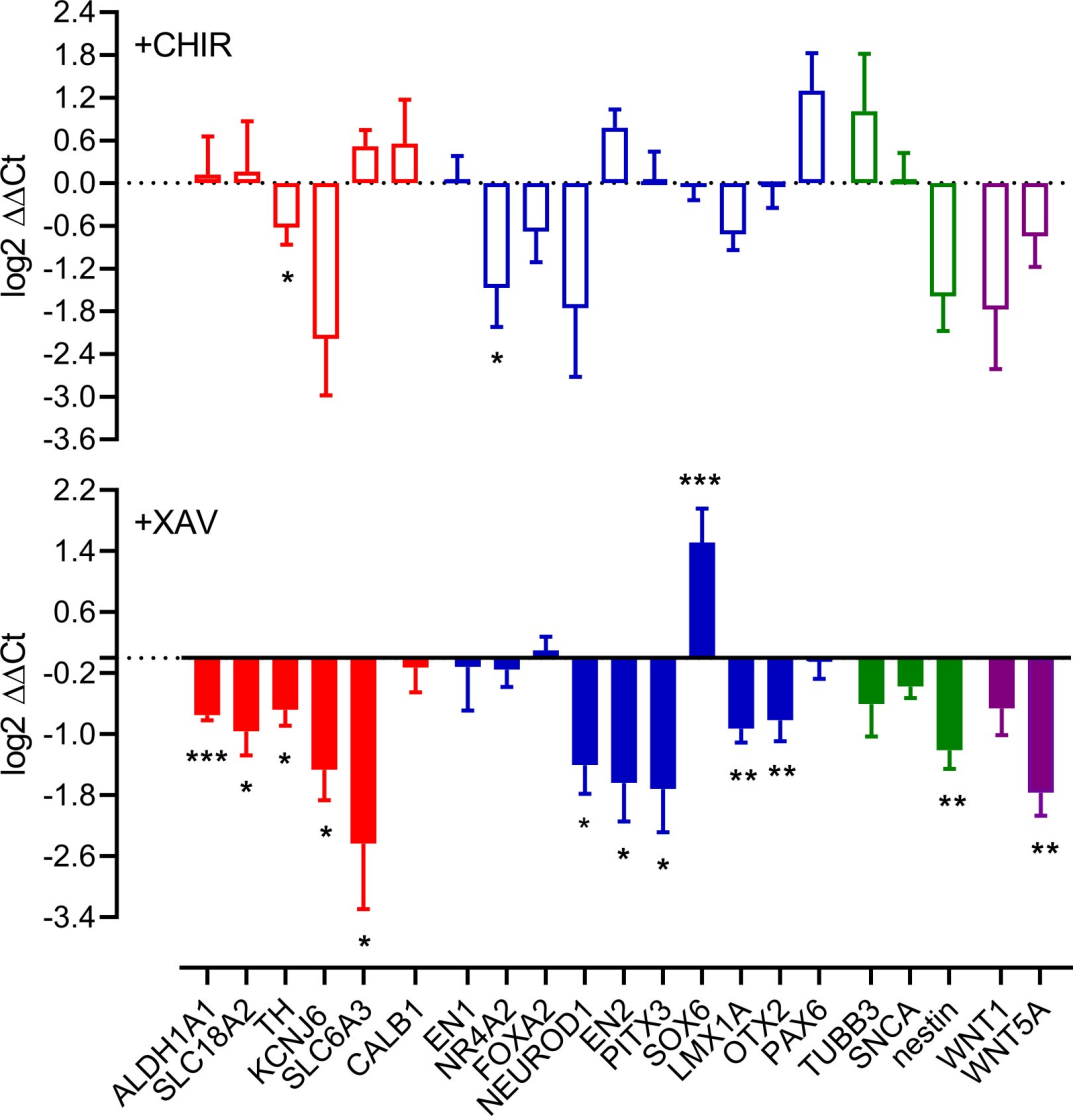
Comparison of dopaminergic gene marker expression at day 65 of differentiation. Data are expressed as log_2_ ΔΔ^Ct^ values for presentation, but statistical analysis was performed using XAV or CHIR vs vehicle control Δ^Ct^ values in either paired t-tests or repeated measures one-ANOVA with post-hoc Dunnett’s tests (n = 3–10). *and ** indicate P<0.05 and <0.01, respectively.

**Fig 3:**
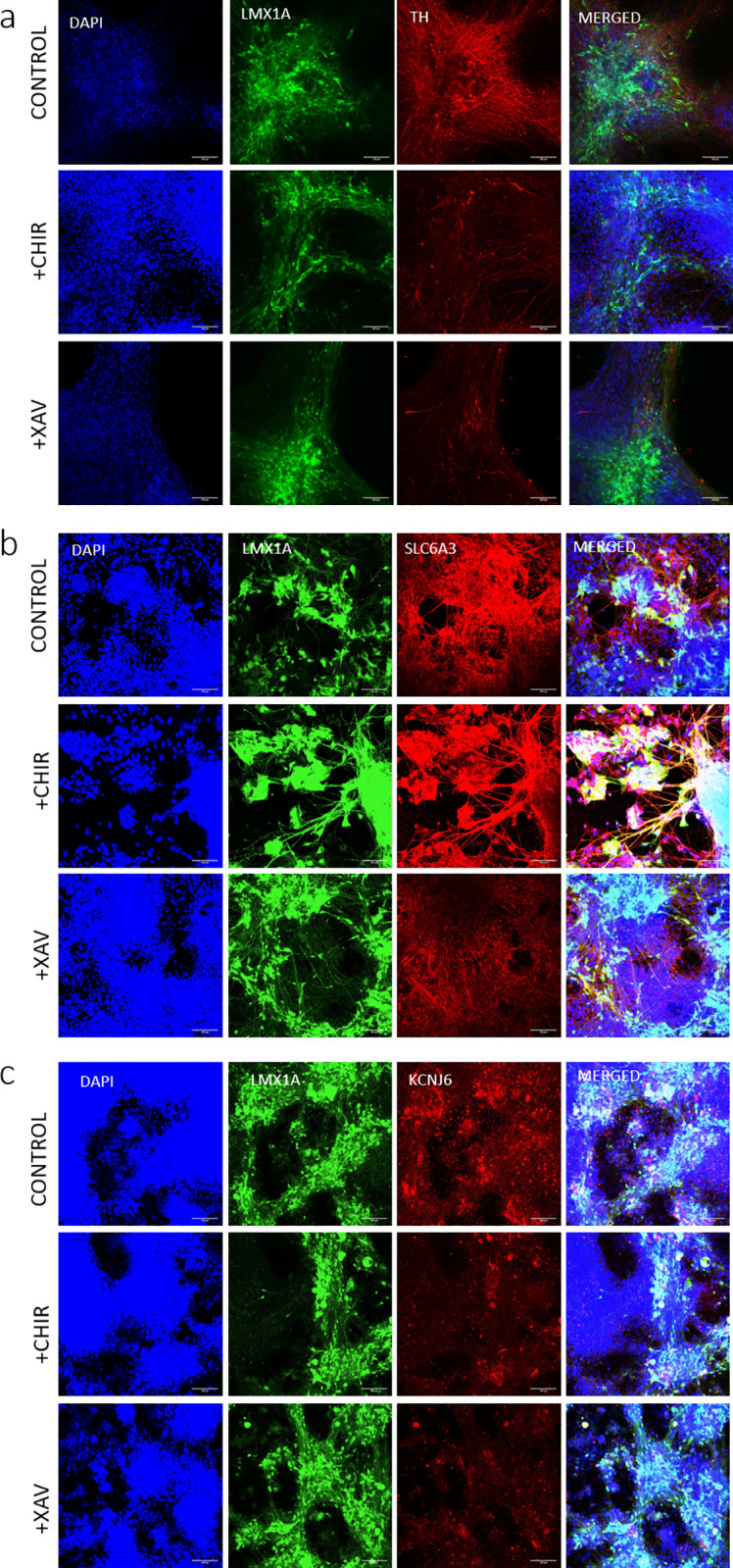
Typical immunolabelling studies showing the impact of CHIR (1μM) and XAV (100nM) upon markers of a dopaminergic neural phenotype. Panel (a) shows tyrosine hydroxylase (TH), panel (b) shows dopamine transporter (SLC6A3) and panel (c) shows potassium inwardly rectifying channel subfamily J member 6 (KCNJ6) in maturing cultures directed toward midbrain differentiation (day 65). Within panels (a), (b) and (c) each of the images, from left to right, show DAPI, LMX1A (eGFP), gene of interest (TH, SLC6A3 or KCNJ6) and a colour combined image. Scale bar indicates 100μm.

We then assessed the impact of the β-catenin-dependent WNT signalling modulators CHIR and XAV upon cultures, late in differentiation during a period of relatively stable gene expression (days 40–65). CHIR (1μM) reduced expression of two gene transcripts associated with dopaminergic neuron phenotype; *NR4A2* and *TH* without greatly impacting others (Fig 2). In contrast, XAV elicited significant reductions in multiple markers of a dopaminergic (and in particular A9) phenotype: *SLC18A2*, Potassium Inwardly Rectifying Channel Subfamily J Member 6 (*KCNJ6* or *GIRK2)*, Aldehyde Dehydrogenase 1 Family Member A1 (*ALDH1A1*), *TH*, Paired Like Homeodomain 3 (*PITX3*), *LMX1A*, Orthodenticle Homeobox 2 (*OTX2)*, and *EN2*. XAV also increased expression of another A9 marker, SRY-Box Transcription Factor 6 (*SOX6*), but did not change expression of the generic neuronal markers β3 tubulin or synuclein. XAV also decreased expression of nestin, and *WNT5A*, but not *WNT1* (Fig 2). Immunolabelling of cultures with antibodies for TH, KCNJ6 and SLC6A3 was consistent with PCR studies, showing some changes following incubations with both CHIR (TH and KCNJ6) and XAV (TH, SLC6A3 and KCNJ6; Fig 3, panels a, b and c, respectively).

Another transcription factor affected by XAV was Neuronal Differentiation 1 (*NEUROD1*), an important regulator of neuronal differentiation expressed within the midbrain area throughout life, and overlapping with TH expression [40].

### Is there a common transcription factor regulating genes affected by XAV?

We turned to MotifMap [37] to look for common putative transcription factor binding sites in the genes whose expression was reduced by XAV (ie. *ALDH1A1*, *SLC18A2*, *TH*, *KCNJ6*, *EN2*, *PITX3 LMX1A*, *OTX2*, *NES* and *WNT5A*, Fig 2). This analysis revealed a short list of putative common transcription factor binding sites (in order of abundance, but not corrected for orientation): NEURO D, LEF1, PDX1 (IPF1), TEAD1, MAFB, ETS2, NR4A2 and STATs (including STAT1,2,3,4,5A & 6). A complete list of putative transcription factor binding sites as indicated by MotifMap is shown in S5 Table.

**Fig 4:**
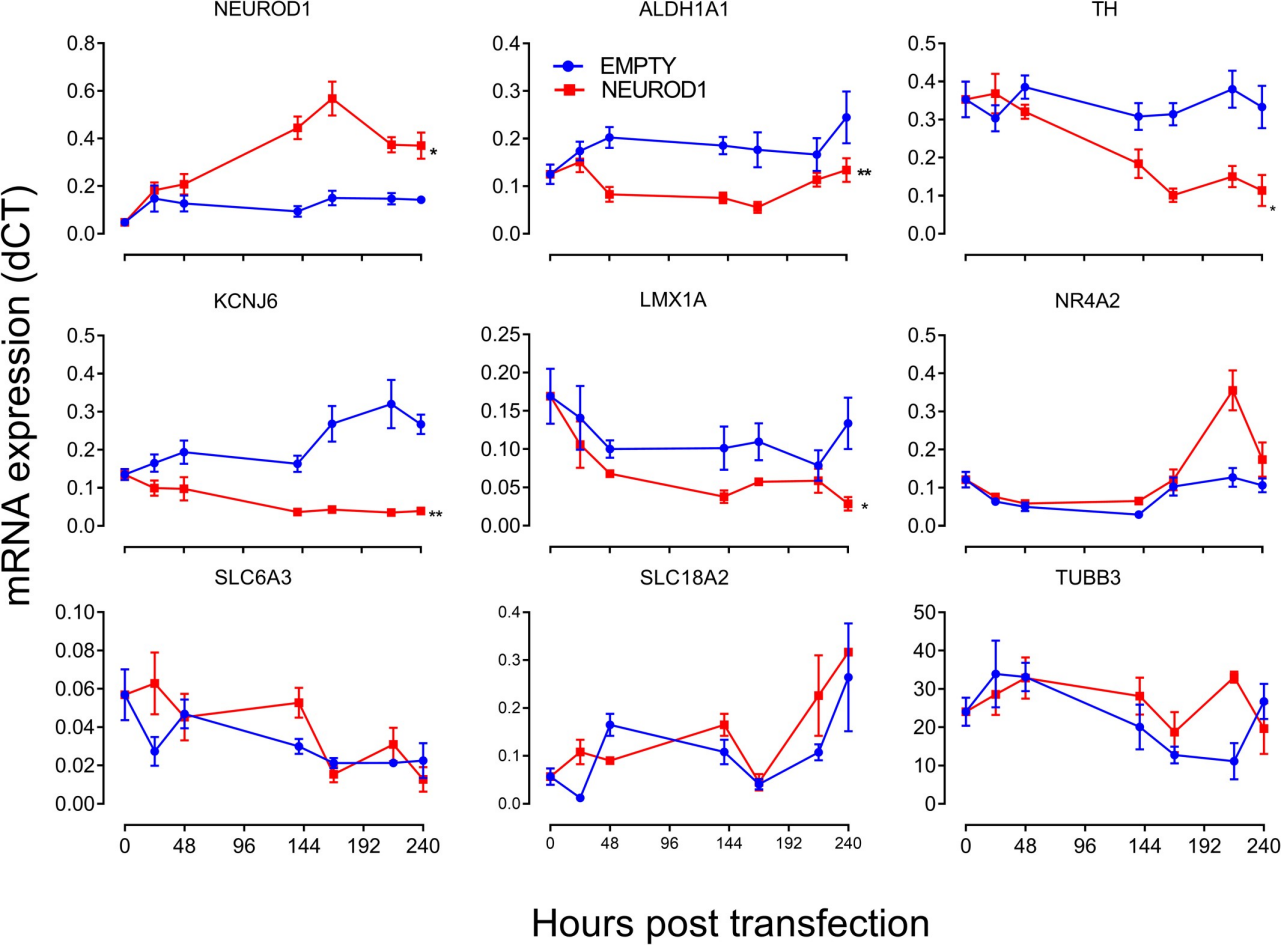
Transfection with NEUROD1 impacts dopaminergic neuronal phenotype. Cultures showed increased *NEUROD1* along with reduced *ALDH1A1*, *TH*, *KCNJ6* and *LMX1A*, but no change in *NR4A2*, *SLC6A3 SLC18A2* or *TUBB3*. Control wells underwent transfection with an empty plasmid. Results are expressed as mean ± SEM of three (biological) replicate experiments. Analysis was performed with t-tests where each post transfection timepoint represented a paired measure (t = 0 was not included in analyses). * and ** indicate P<0.05 and P<0.01, respectively. S1 Fig shows NEUROD1 immunolabelling following transfection.

While the preponderance of putative LEF1 transcription factor binding sites in genes affected by XAV was reassuring, some genes showing profound effects, namely *EN2* and *PITX3* possessed few putative LEF1 transcription factor binding sites but were rich with putative NEURO D transcription factor binding sites. That XAV (100nM) reduced NEUROD1 expression in cultures (Fig 2) is therefore consistent with the idea that a loss of NEUROD1 signalling contributes to a loss of dopaminergic neuronal phenotype. Given the early and persistent expression of NEUROD1 in mouse dopaminergic neuron differentiation and maturation [40], we began with the idea that NEUROD1 overexpression might serve to rescue dopaminergic phenotype under the influence of XAV signalling. Surprisingly, our initial experiments showed that following transfection, cultures overexpressing *NEUROD1* showed reduced *ALDH1A1*, *TH*, *KCNJ6* and *LMX1A*, along with no change in *NR4A2*, *SLC6A3 SLC18A2* or *TUBB3* transcript (Fig 4; immunolabelling showing increased NEUROD1 protein is shown in S1 Fig). Essentially, this data indicates that while neuronal phenotype was largely unaffected, NEUROD1 reduced expression of markers of an A9 dopaminergic neuron phenotype (i.e. *KCNJ6*, *ALDH1A1* and *TH*). These data are more in agreement with the findings of Park et al., [41] that NEUROD1 opposes the impact of NR4A2, rather than Val Cervo et al., [42] who showed that NEUROD1 promoted a dopaminergic neuronal phenotype. Although, our data indicate that overexpression of NEUROD1 reduces dopaminergic, and in particular, an A9 phenotype we also saw that NEUROD1 overexpression did not affect *WNT1* or *WNT5A* expression (not shown). Given that NEUROD1 is widely regarded as a transcriptional activator, we searched the literature for potential mechanisms that might enable NEUROD1 to inhibit expression of *ALDH1A1*, *KCNJ6*, *TH* and *LMX1A*. We identified SHP1 (small heterodimer partner 1, *NR0B2*), a corepressor of NEURO D (NEUROD1) that competes with coactivator p300 for binding sites [43], and another repressor protein DAX (*NR0B1*) [44] that represses the activity of another gene; hepatocyte nuclear factor 4 (HNF4) [45, 46], previously identified as a potential biomarker for PD [47].

**Fig 5:**
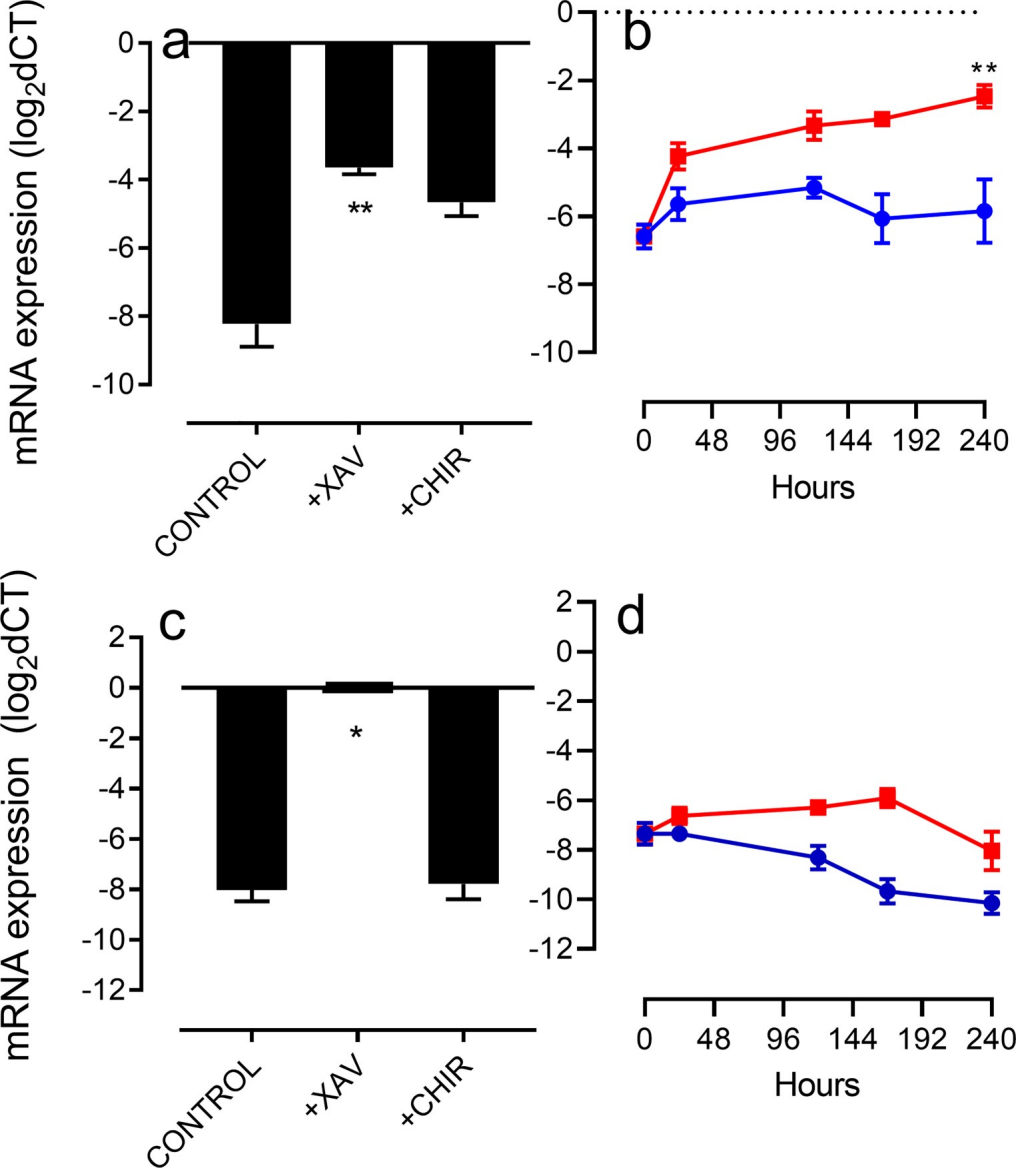
Changes in *NR0B1* and *NR0B2* transcript expression following incubation with CHIR (1μM) or XAV (100nM) or transfection to overexpress NEUROD1. Panels (a) and (b) respectively show the effects of the WNT modulators and NEUROD1 overexpression upon *NR0B1*. Panels (c) and (d) show effects upon *NR0B2* expression. Results are expressed as mean ± SEM of three (biological) replicate experiments. Analysis of panel (a) was via one-way ANOVA with post-hoc Dunnett’s test. For panels (b) and (d) analysis was via Student’s t-tests (at each time point as in Fig 4). For panel (c) analysis was via a non-parametric Kruskal-Wallis test with post-hoc Dunn’s test to accommodate undetectable levels of *NR0B2* in two out of three samples, both undetectable values were assigned to 0.0028 (equal to the lowest value detected).

**Fig 6:**
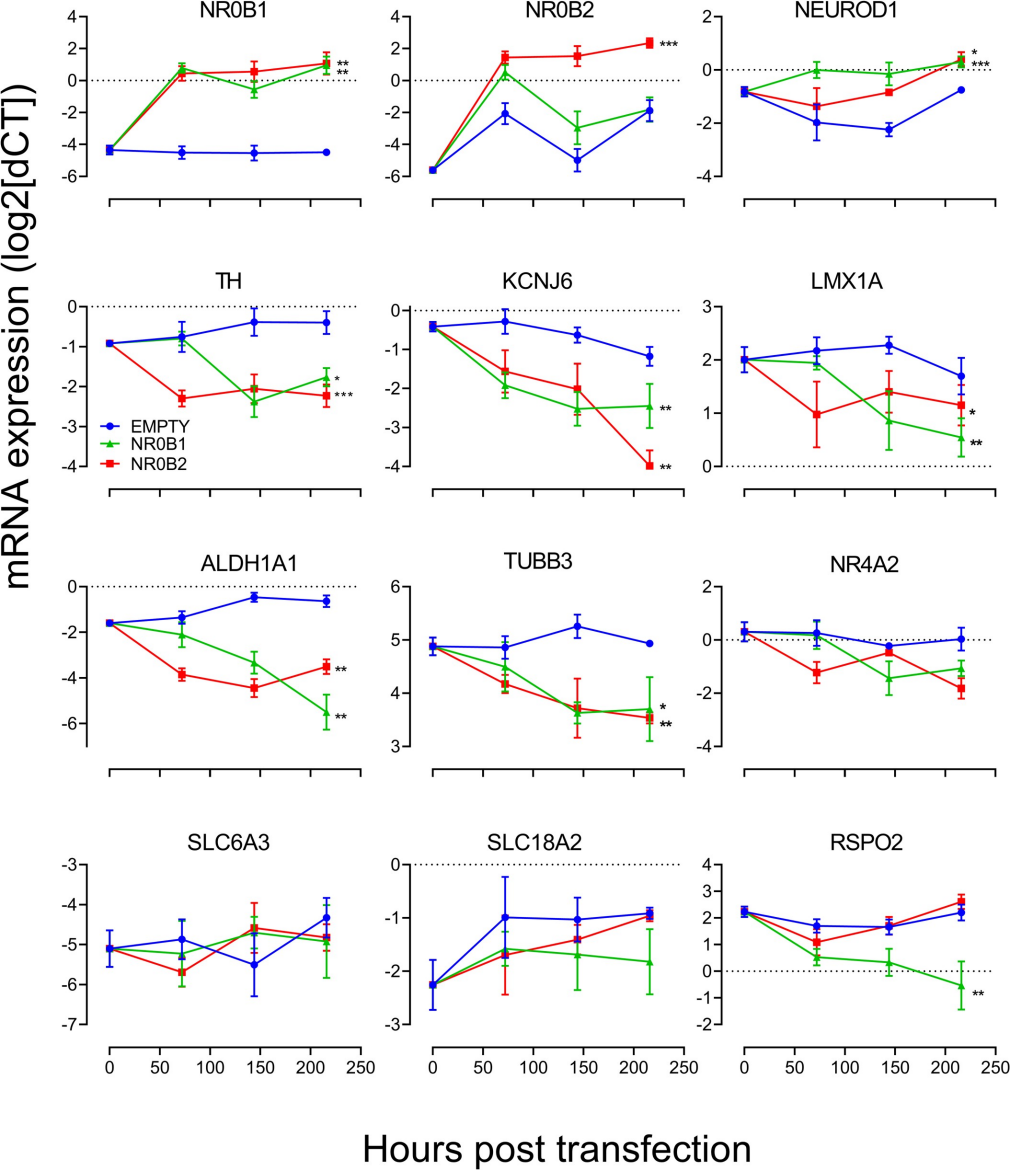
Transfection with either NR0B1 and NR0B2 impacts dopaminergic neuronal phenotype. Expression patterns following transfection with *NR0B1* (green), *NR0B2* (red) or blank (blue) plasmids show that NR0B1 transfection increases both *NR0B1* and *NR0B2* and impacts expression of *NEUROD1*, *TH*, *KCNJ6*, *LMX1A* and *TUBB3*. Control wells underwent transfection with an empty plasmid. Results are expressed as mean ± SEM of three (biological) replicate experiments. Analysis was performed with One-way ANOVA followed by post-hoc Dunnett’s test where each post transfection timepoint represented a repeated measure (t = 0 was not included in analyses). * and ** indicate P<0.05 and P<0.01, respectively. S3 Fig shows increased NR0B1 and NR0B2 immunolabelling up to six days after transfection.

We assessed the activity of XAV and CHIR in regulating *NR0B1 & NR0B2* expression and found that XAV, but not CHIR, significantly elevated both transcripts (Fig 5, panels (a) and (c)). Consistent with the impacts upon transcript expression, NR0B1 and NR0B2 proteins were largely undetectable unless neurons were incubated with XAV (S2 Fig, panels a and b respectively). In contrast with the impact of XAV, overexpression of NEUROD1 promoted significant elevation of *NR0B1* but not *NR0B2* (Fig 5, panels (b) and (d)). These data show that over a 25-day incubation, the addition of XAV correlates with increased *NR0B1/2*, but major reductions in both *NEUROD1* and A9 phenotype markers. In contrast, an elevation of *NEUROD1* results in elevated *NR0B1* but also reduced expression of A9 phenotype markers. These data led us to speculate that NR0B1 is a critical regulator of dopaminergic neuronal phenotype so we investigated the idea that NR0B1 could directly impact expression of dopaminergic markers. We overexpressed *NR0B1* and *NR0B2* in mature midbrain cultures and found that overexpression of *NR0B1* significantly upregulated expression of both *NR0B1* and *NR0B2* (Fig 6) while promoting significant reductions in a number of key dopaminergic neuronal phenotype markers; *TH*, *KCNJ6*, *ALDH1A1* and *LMX1A*, but not *SLC6A3*, *SLC18A2* or *NR4A2*. Curiously *TUBB3* was profoundly downregulated by both *NR0B1* and *NR0B2* overexpression (Fig 6). Consistent with changes in transcript *NR0B1* and *NR0B2* overexpression increased expression of NR0B1 and NR0B2 (S3 Fig, panels a and b, respectively).

### How are *NEUROD1* and *NR0B1* and *NR0B2* impacted in Parkinson’s disease?

**Fig 7:**
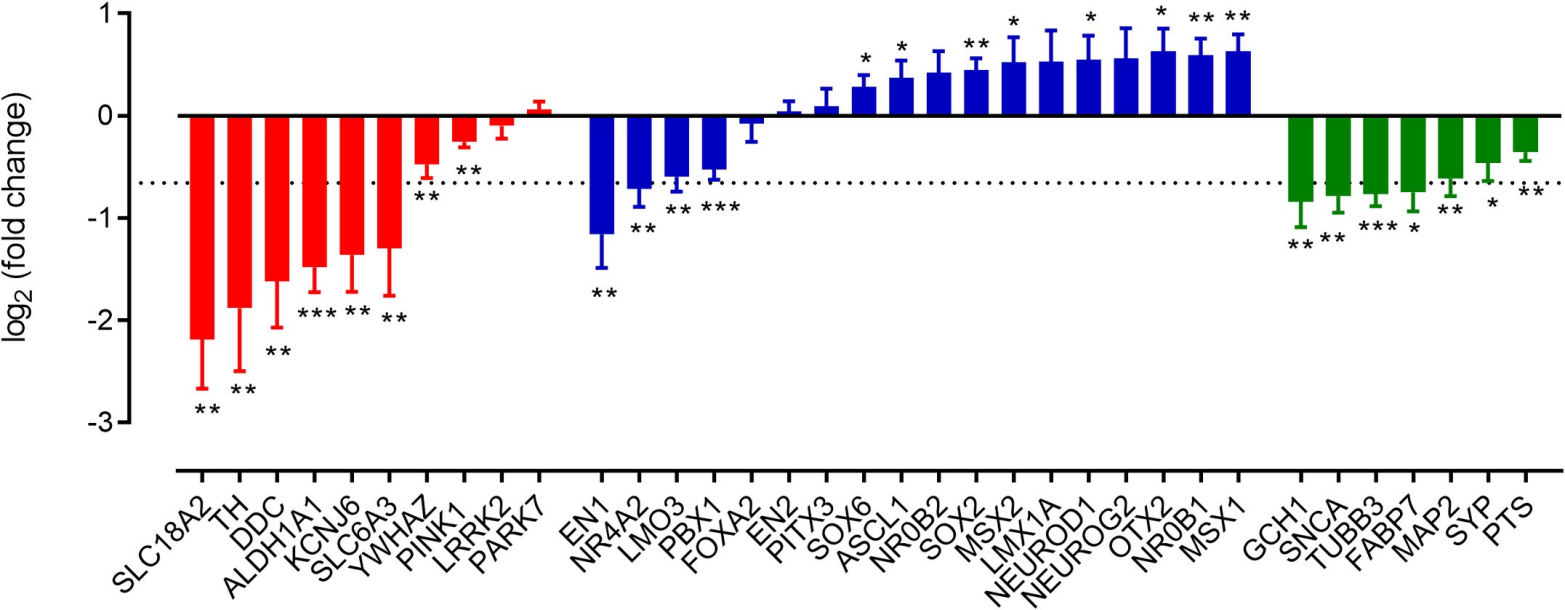
PD-related changes in expression of key markers of neuronal phenotype and dopaminergic neuronal development. The initial group (red) shows transcripts whose loss is usually correlated with PD, for example TH and SLC6A3. The middle group (blue) shows how PD impacts upon developmental and mature neuron transcripts. The right-hand grouping (green) shows neuronal markers, rather than dopaminergic neuronal markers. The dotted line indicates the average loss of neuronal markers. The Δ^Ct^ values for each control and PD transcript were analysed using Student’s paired t-tests. *, ** = P<0.05, 0.01, respectively (n = 8–10 datasets).

We found the idea of a specific regulation of dopaminergic neuron identity fascinating. So we assessed PD array databases, initially looking at the genes defining dopaminergic neuron development and phenotype (such as *FOXA2*, *LMX1A*, *EN1*, *EN2*, *SLC18A2*, *TH*, *DDC*, *ALDH1A1*, *NR4A2*, *PITX3*, *SOX6*) as well as more generic markers of a neuronal phenotype (*SNCA*, *SYP*, *FABP7*, *GCH1*, *PTS*, *TUBB3*, *MAP2*). This analysis showed loss of multiple neuronal transcripts: *SNCA*, *SYP*, *FABP7*, *GCH1*, *PTS*, *TUBB3*, *MAP2* (Fig 7, in green) alongside losses of transcripts associated with dopaminergic neuronal function (*SLC18A2*, *TH*, *ALDH1A1*, *KCNJ6* Fig 7, in red) and smaller changes in other transcripts associated with a dopaminergic neuronal phenotype, such as *EN1*, *NR4A2*, *LMO3 and PBX1* (Fig 7, in blue). Curiously, some transcripts were unaffected by PD (*FOXA2*, *LMX1A*, *PITX3* and *NR0B2*) while some; *MSX1*, *MSX2*, *ASCL1*, *OTX2*, and most importantly, *NEUROD1* and *NR0B1* were all significantly elevated. We used human tissues from control and individuals with PD to check known dopaminergic and midbrain markers protein expression levels and found PITX3/TH immunolabelling (S4 Fig) to be broadly consistent with transcriptomic data.

**Fig 8:**
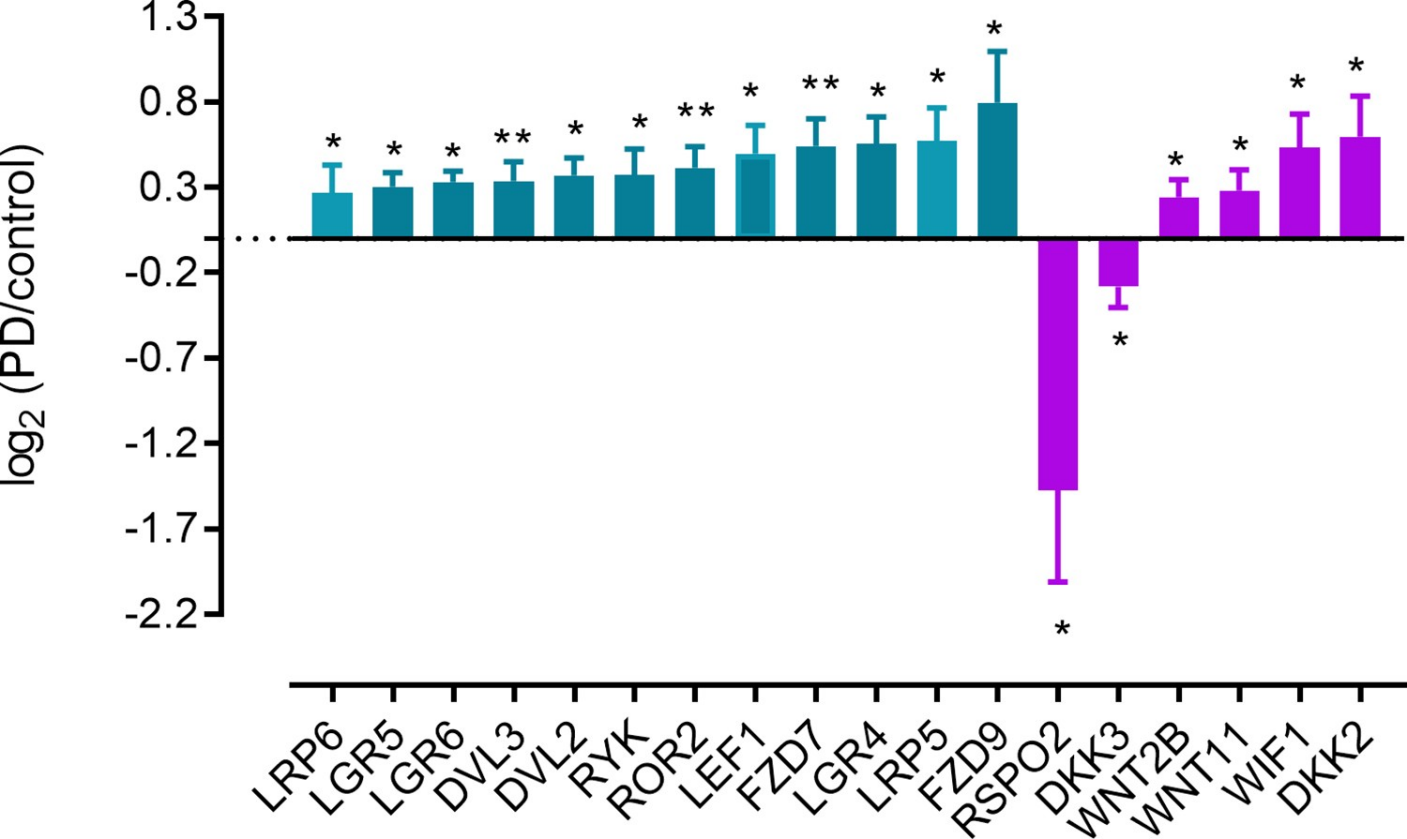
Significant changes in WNT signalling transcripts associated with PD. Membrane bound components increased (teal) while soluble factors increased and decreased (magenta). Analysis is identical that in Fig 3.

Given the impact that a loss of β-catenin-dependent WNT signalling has upon dopaminergic neuron markers *in vitro*, we evaluated the PD databases for changes in soluble and membrane bound WNT signalling components. Thus, we looked for changes in frizzled receptors, leucine rich repeat containing G-protein coupled receptors (*LGR4*, *5 & 6*) and LDL receptor related proteins (*LRP4*, *5 & 6*), *Kremen1* & *Kremen2*, *ROR1 & ROR2* and *RYK*. We also looked for changes in soluble WNT signalling regulators (WNT-inhibitory factor (*WIF1*), secreted frizzled related proteins (*SFRP1*, *2*, *4 & 5*), dickkopfs (*DKK1*, *2*, *3 & 4*), disheveled (*DVL1*, *DVL2*, *DVL3*) and the R-spondins (*RSPO1*, *2*, *3 & 4*). This analysis showed some profound changes, particularly a strong reduction of *RSPO2*, a gene encoding the secreted protein roof plate-specific spondin-2, an activator of β-catenin-dependent WNT signalling (Fig 8 summarises the statistically significant, PD-associated changes in WNT signalling regulators). All changes in WNT signal-related transcript are shown in S5 Fig. Significantly, we found that overexpression of *NR0B1*, but not *NR0B2* promoted reduction in *RSPO2* expression from cultures (Fig 6).

One particularly interesting element of this study is the finding that the absence of β-catenin-dependent WNT signalling (XAV) promoted a profound elevation of *SOX6* transcript expression. This is noteworthy as there are no putative LEF1 binding sites and very few putative NEURO D binding sites regulating transcription (see S5 Table), which may indicate additional mechanisms underlying gene regulation.

## Discussion

Transplantation therapies for PD rely upon appropriately patterned dopaminergic neural progenitors to supplement dopamine production from the surviving A9 neurons. Progenitors at or before day 20 of differentiation are used for transplantation since they offer increased connectivity and integration with host neurons as they mature (for reviews see [23, 24]). The use of these cells is predicated on the assumption that following patterning, dopaminergic progenitors do not undergo substantive changes after transplantation. Protocols for creating midbrain neural progenitors, *in vitro*, draw heavily from our understanding of mouse neuronal differentiation and rely upon sonic hedgehog and WNT signalling to regulate development (see [5, 6, 8, 48]) as defined by expression of transcription factors such as *EN1/2*, *OTX2*, *FOXA2*, *LMX1A*, *MSX1*, *NEUROG2* and later, *ASCL1*, *NR4A2*, *PITX3* [5–9] and *PBX1* [10]. Within the adult midbrain, dopaminergic neurons of the substantia nigra (A9) and the adjacent ventral tegmental area (A10) are both characterized by expression of TH: markers such as aldehyde dehydrogenase (ALDH1A1, [13]), KCNJ6 (G Protein-Activated Inward Rectifier Potassium Channel 2, [14]), the calcium binding protein, calbindin (CALB1), orthodenticle homeobox 2 (OTX2) and SRY (Sex Determining Region Y)-Box 6 (SOX6, see [15]) are subsequently used to define A9 (ALDH1A1, KCNJ6, SOX6) and A10 (OTX2, CALB1) neurons.

Over the course of 80 days our maturing cultures showed expression of markers consistent with the development and maturation of dopaminergic neurons; early markers such as FOXA2 and LMX1A decrease (but do not disappear) after day 20 of differentiation as markers of a more mature dopaminergic neuronal phenotype such as TH, SLC18A2 and synuclein increase and plateau. These findings are broadly consistent with changes in gene expression in late developing cultures identified previously [36]. However, a key consideration in understanding how developing dopaminergic cultures develop and maintain a phenotype is that, at least in mouse development, early genes/patterning factors do not necessarily “switch off”, but remain expressed in post-mitotic neurons (see [12]). This idea provides the platform for the current study. Recent work has shown that excessive activation of the β-catenin-dependent WNT pathway results in gene expression changes of midbrain floorplate progenitors and decreased numbers of mDA neurons. Interestingly, this work also shows a severe reduction of dopaminergic neurons at the rostral midbrain region (location of A9 neurons), whilst the caudal midbrain regions (location of A10 neurons) were not affected [49]. This finding supports the idea that A9 and A10 neurons may be regulated, in part, by β-catenin-dependent WNT signalling [50]. With this in mind we investigated the impact of CHIR, a commonly used inhibitor of glycogen synthase kinase 3β (GSK3β) which, along with other sonic hedgehog signalling mimetics, is used as a driver of dopaminergic differentiation [35, 51–53]. We also investigated the impact of XAV939, a tankyrase inhibitor that antagonizes β-catenin-dependent WNT signalling [54], which is used drive cortical differentiation [55]. We opted to assess the impact of WNT signalling upon cultures that had achieved relative stability, ie after day 40 when transcripts for TH, LMX1A and TUBB3 had largely stabilized, thus we added WNT signalling modulators from days 40 to 65 of maturation. CHIR reduced expression of two gene transcripts commonly associated with dopaminergic neuron maturation; *NR4A2* and *TH*, indicating a very subtle shift away from a dopaminergic phenotype following 25 days of activation. In comparison, XAV significantly reduced expression of many genes associated with a midbrain dopaminergic neuronal phenotype including *SLC18A2*, *KCNJ6*, *ALDH1A1*, *TH*, *PITX3*, *LMX1A*, *OTX2*, and *EN2*. In support of these findings, XAV and to a lesser extent, CHIR, appeared to reduce the intensity of TH, KCNJ6 and SLC6A3 immunolabeling. XAV did not, however, affect markers of a neuronal phenotype (β3-tubulin or synuclein) indicating a shift away from a dopaminergic neuronal phenotype (rather than a neuronal phenotype). That the proposed markers of an A9 phenotype: SOX6 [56], KCNJ6 (GIRK2 [16]) and ALDH1A1 [17] showed different expression patterns following XAV raises questions around the choice of a single marker to identify A9 neurons. The decreased nestin expression in response to XAV might implicate β-catenin-dependent WNT signalling in regulating neural progenitor growth [57], although nestin has also been shown to positively regulate β-catenin-dependent WNT signalling as well [58], suggesting a positive feedback loop. Confounding this is the finding of a XAV-mediated reduction of WNT5A transcript. While Wnt5a plays a role in mouse dopaminergic neuron development [8], this finding may indicate an active regulatory WNT signalling feedback loop present in cultures [59]. As the activation of β-catenin-dependent-WNT signalling with CHIR in our system has little effect, but inhibiting that system has a profound influence, we suggest that cultures possess active β-catenin-dependent-WNT signalling pathways that provide ongoing maintenance of dopaminergic phenotype. While our data shows a dynamic regulation of WNT5A transcript, other WNT signalling regulators such as dickkopf 3 [19] and Rspo2 (r-spondin 2) (86) have been shown to regulate dopaminergic neuron development and phenotype. At present the nature of the ligands contributing to WNT signalling to regulate dopaminergic neuron phenotype in culture is unclear.

One particularly interesting finding was that XAV significantly reduced the expression of NEUROD1; a member of the basic helix-loop-helix (bHLH) family of transcription factors linked to neurogenesis [60]. It is expressed in the adult murine substantia nigra [40] and while overexpression of NeuroD diminishes the capacity of Nr4a2 to drive expression of Th, dopamine transporter, Vmat2 and dopa decarboxylase in rat neural precursor cells [41] it has been used as one of four factors to reprogram astrocytes to dopaminergic neurons [42]. Our data show that *NEUROD1* transcript expression is reduced by XAV, which is consistent with evidence that NeuroD1 expression is upregulated by β-catenin-dependent WNT signalling (upon removal of Sox2 mediated transcriptional repression [21]). Given the role of NeuroD1 in reprogramming astrocytes to dopaminergic neurons [42] we surmised that overexpression of NEUROD1 regulate markers of a dopaminergic neuronal phenotype. In contrast to expectations NEUROD1 overexpression reduced markers of an A9 dopaminergic neuron phenotype. That it is specific for a handful of genes, but does not correlate with the distribution of MotifMap identified putative NEUROD transcription factor binding sites indicates a complex relationship between NEUROD1 expression and dopaminergic (and in particular A9) phenotype. The nature of this relationship might be clarified by considering the role that the transcriptional repressors, SHP1 (*NR0B2*) and DAX (*NR0B1*) play in regulating gene expression in cultured neurons. While XAV reduced *NEUROD1* it profoundly elevated both *NR0B1* and *NR0B2* expression. NR0B1 and NR0B2 can inhibit expression of not only NEUROD1 regulated genes [43], but also HNF4 regulated genes [45, 46], and MotifMap analysis indicates an abundance of putative HNF4 regulatory elements controlling dopaminergic neuronal phenotype. That both XAV as well as NEUROD1 overexpression promoted elevations of both NR0B1 and NR0B2 may be consistent with a loss of the ability of NEUROD1, and perhaps HNF4, to drive expression of genes related to dopaminergic neuronal phenotype. Couple this with a XAV driven loss of β-catenin-dependent WNT signalling (ie. reduced LEF1 activity) and it is perhaps not surprising that there is a loss of dopaminergic neuronal phenotype in culture. While *NEUROD1* overexpression elevates *NR0B1* it is worth noting that *NR0B1* overexpression drives *NR0B2* and *NEUROD1* (possibly indicating the presence of a feedback loop). Thus, over expression of *NR0B1* and *NR0B2* is capable of selectively supressing *TH* and *LMX1A* as well as markers of an A9 dopaminergic neuronal phenotype (*ALDH1A1* and *KCNJ6)*. Curiously we also show that *NR0B1* and *NR0B2* overexpression reduces expression of *TUBB3* (which has an abundance of putative NEUROD transcription factor binding sites), a finding that may indicate suppression or degradation of axon guidance and/or maintenance.

One curious finding of this study was the XAV-mediated increase in *SOX6* expression. Sox2 and Sox6 form a positive feedback loop to regulate neuronal differentiation [61] and decreasing Sox6 expression in 3T3L1 cells increases levels of β-catenin [62]. Exactly how *SOX6* is elevated by XAV is unclear, since there are few putative NEUROD1 or LEF1 transcription factor binding sites regulating expression, indeed MotifMap analysis indicates the regulation of *SOX6* expression to be quite distinct from the other genes of interest, including *NR0B1* and *NR0B2*.

Given our evidence showing that inhibition of β-catenin-dependent WNT signalling reduced a number of markers of an A9 midbrain dopaminergic phenotype while increasing *NR0B1* and *NR0B2* expression, we wondered whether WNT signalling systems could be perturbed in PD. We initially assessed PD array databases (GEO accession numbers: GSE20163, GSE20164, GSE20141 [27], GSE20164 [28], GSE20333 (Edna et al., [unpublished]), GSE7307 [29], GDS3128 [30], GDS3129 [30], GSE54282 [31], GSE43490 [32]) to look at changes in genes defining dopaminergic neuron development and phenotype as well as more generic markers of a neuronal phenotype. Consistent with the idea that PD is associated with the loss of dopaminergic neurons, this analysis showed significant and consistent loss of transcripts for multiple neuronal genes: *SNCA*, *SYP*, *FABP7*, *GCH1*, *PTS*, *TUBB3*, *MAP2*. We also showed very profound and expected loss of transcripts associated with dopaminergic neuronal function (*SLC18A2*, *TH*, *ALDH1A1*, *KCNJ6*), but smaller losses of other transcripts associated with a dopaminergic neuronal phenotype, such as *EN1*, *NR4A2*, *LMO3* and *PBX1*. A most curious finding was that a number of genes associated with a dopaminergic phenotype (for example, *FOXA2*, *EN2*, *LMX1A* and *PITX3*) did not show reductions in transcript, while some; *OTX2*, *MSX1/2 ASCL1*, *NEUROD1*, *SOX2*, *SOX6*, and *NR0B1* increased. We then looked for changes in WNT signalling components in the PD array databases, reasoning that changes in WNT signalling transcripts might provide a clue to the regulation of the genes showing such a profound depression in PD (ie. *SLC18A2*, *TH*, *ALDH1A1*, *SLC6A3* and *KCNJ6*). We looked for changes in soluble and membrane bound components of the WNT signalling cascade and found a significant upregulation of some cellular and soluble components of the WNT signalling cascade, most notably a profound loss of RSPO2 transcript. R-spondins are secreted ligands of leucine-rich repeat containing G protein-coupled receptors that enhance WNT signalling [63]. Consistent with the idea of a loss of β-catenin-dependent WNT signalling is a PD associated increase in multiple membrane bound WNT signalling components (*RYK*, *ROR2*, *LGR5*, *LGR5*, *FZD7*, *FZD9*), along with increased *WNT1*, *2B* and *11*, as well as *WIFI* and *DKK2*. Interestingly *DKK3* transcript was also reduced; DKK3 plays a role in midbrain dopaminergic neuron development [19]. Supporting the idea that WNT signalling may be perturbed in PD we identified that both *NEUROD1* and *NR0B1* are significantly upregulated. Since, in culture, overexpression of *NEUROD1* promotes expression of *NR0B1* and since *NR0B1* overexpression reduces markers of a dopaminergic, and in particular an A9 phenotype, as well as reducing *RSPO2* expression, we expect that elevated nigral *NEUROD1* and *NR0B1* expression would induce the same effect, ie. a reduction in the appearance of A9 markers along with a reduction in *RSPO2* expression. This hypothesis clearly needs further investigation.

We believe that our *in vitro* model data support the idea that at least a proportion of the genes essential for the characterization of dopaminergic phenotype are regulated by β-catenin-dependent-WNT signalling either directly by *NEUROD1* expression, or indirectly the elevation of *NR0B1* (possibly working in conjunction with *NR0B2*). Confounding our efforts to identify genes regulated by XAV and or *NEUROD1/NR0B1/NR0B2* overexpression is that multiple signalling systems are activated or inhibited by the presence of the neurotrophic factors added to cultures; these include GDNF, BDNF, TGFβ3, dbcAMP as well as the notch inhibitor, DAPT. These factors can regulate expression of many genes identified in this study, including MAFB, PDX1, TEF-1, CREB and ETS2 [64–71]. In addition, while our culture protocol enriches for dopaminergic neurons, both forebrain and hindbrain markers are present in differentiations [36]. Whether and or how these other cell types contribute to WNT signalling is currently unknown.

In summary, we have shown that WNT signalling plays an ongoing role in regulating dopaminergic neuronal phenotype, even late in maturation. As part of this process the loss of β-catenin-dependent WNT signalling in cultures profoundly increases expression of the transcriptional repressors, *NR0B1/2* which in-turn downregulate dopaminergic, and in particular A9 neuronal markers, as well as *RSPO2*. The PD array database profiling indicates that tissue of the substantia nigra has reduced *RSPO2* and elevated *NR0B1* (and *NEUROD1)* leading us to believe that dysregulation of β-catenin-dependent WNT signalling is evident in the substantia nigra of individuals with PD. While this dysregulation may exacerbate the degeneration-induced loss of dopaminergic neurons we believe that, regardless of the site of transplantation, local brain chemistry will have a profound impact upon the ability of transplanted neural progenitors to maintain a dopaminergic neuronal phenotype.

## Supporting information

S1 Table GEO datasets used in this study. (DOCX)

S2 Table Taqman probes used for quantitative PCR. (DOCX)

S3 Table PCR data. (XLSX)

S4 Table Primary and secondary antibodies used in this study. (DOCX)

S5 Table MotifMap analysis of putative transcriptional control. Genes of interest are listed at the top of each column. Pairs of columns show transcription factor and putative number of binding sites. Sites were estimated using MotifMap (http://motifmap.ics.uci.edu/). Note: Putative binding sites are not corrected for overlap or orientation.
(XLSX)

S1 Fig Post-NEUROD1 transfection immunolabelling. Panels (a)-(e) show Hoechst nuclear labelling, LMX1A-eGFP, NEUROD1, β3-tubulin and colour combined images seven days after NEUROD1 transfection. Panels (f)-(j) show Hoechst nuclear labelling, LMX1A-eGFP, NEUROD1, β3-tubulin and colour combined images seven days after control transfection. Images are all 20x (scale bar is 100μm) taken with the same imaging parameters. The insets show x4 images.
(TIF)

S2 Fig NR0B1 and NR0B2 expression following incubation of midbrain cultures with XAV. From left to right the top pair of panels show DAPI nuclear labelling, LMX1A-eGFP, NR0B1, β3-tubulin and colour combined images after 14 days of vehicle (control, upper) or XAV (100nM, lower). The bottom pair of panels show identical labelling except where NR0B2 is indicated. Images are all 20x (scale bar is 100μm) taken with the same imaging parameters.
(TIF)

S3 Fig Transfection with *NR0B1* and *NR0B2* increases protein expression. From left to right the panels DAPI nuclear labelling, LMX1A-eGFP, NR0B1 (top three panels) & NR0B2 (bottom three panels) and colour combined images in vehicle (control, 6 days) and at 3 and 6 days after transfection. Images are all 20x (scale bar is 100μm) taken with the same imaging parameters across each of the days.
(TIF)

S4 Fig Typical PD and control immunolabelling. Panels show tyrosine hydroxylase (green, panels a and e) and PITX3 (red, panels b and f) in sections of substantia nigra from an age-matched control (panels a-d) and a PD patient (panels e-h). Panels (c) and (g) show Hoechst labelled nuclei, while panels (d) and (h) show colour combined images. Note the widespread reduction in green fluorescence in PD. Scale bar indicates 25μm.
(TIF)

S5 Fig PD-related changes in WNT signalling components. Panel (a) shows changes in nigral WNTs, panel (b) shows changes in soluble WNT signalling ligands while panels (c) and (d) show changes in frizzled receptors and cellular WNT-regulators or signal transduction components. Although data is shown as a ratio PD/control, the Δ^Ct^ values for matched control and PD transcript arrays analysed using Student’s paired t-tests. *, ** = P<0.05, 0.01, respectively (n = 6–10 datasets).
(TIF)
