# Supplementary material for: Inhibition of β-catenin dependent WNT signalling upregulates the transcriptional repressor NR0B1 and downregulates markers of an A9 phenotype in human embryonic stem cell-derived dopaminergic neurons: Implications for Parkinson’s disease
Source: PLoS One. 2021 Dec 23;16(12):e0261730. doi: 10.1371/journal.pone.0261730 (PMC8700011; doi:10.1371/journal.pone.0261730)
Supplement: S1 Table — (DOCX) [file pone.0261730.s001.docx]

[GSE20163](https://www.ncbi.nlm.nih.gov/geo/query/acc.cgi?acc=GSE20163) (using datasets [GSM506013](https://www.ncbi.nlm.nih.gov/geo/query/acc.cgi?acc=GSM506013), [GSM506014](https://www.ncbi.nlm.nih.gov/geo/query/acc.cgi?acc=GSM506014), [GSM506015](https://www.ncbi.nlm.nih.gov/geo/query/acc.cgi?acc=GSM506015), [GSM506016](https://www.ncbi.nlm.nih.gov/geo/query/acc.cgi?acc=GSM506016), [GSM506017](https://www.ncbi.nlm.nih.gov/geo/query/acc.cgi?acc=GSM506017), [GSM506018](https://www.ncbi.nlm.nih.gov/geo/query/acc.cgi?acc=GSM506018), [GSM506019](https://www.ncbi.nlm.nih.gov/geo/query/acc.cgi?acc=GSM506019), [GSM506020](https://www.ncbi.nlm.nih.gov/geo/query/acc.cgi?acc=GSM506020), [GSM506021](https://www.ncbi.nlm.nih.gov/geo/query/acc.cgi?acc=GSM506021), [GSM506022](https://www.ncbi.nlm.nih.gov/geo/query/acc.cgi?acc=GSM506022), [GSM506002](https://www.ncbi.nlm.nih.gov/geo/query/acc.cgi?acc=GSM506002), [GSM506004](https://www.ncbi.nlm.nih.gov/geo/query/acc.cgi?acc=GSM506004), [GSM506005](https://www.ncbi.nlm.nih.gov/geo/query/acc.cgi?acc=GSM506005), [GSM506006](https://www.ncbi.nlm.nih.gov/geo/query/acc.cgi?acc=GSM506006), [GSM506009](https://www.ncbi.nlm.nih.gov/geo/query/acc.cgi?acc=GSM506009), [GSM506010](https://www.ncbi.nlm.nih.gov/geo/query/acc.cgi?acc=GSM506010), [GSM506011](https://www.ncbi.nlm.nih.gov/geo/query/acc.cgi?acc=GSM506011), [GSM506012](https://www.ncbi.nlm.nih.gov/geo/query/acc.cgi?acc=GSM506012)).

[GSE20141](https://www.ncbi.nlm.nih.gov/geo/query/acc.cgi?acc=GSE20141) (using datasets [GSM503950](https://www.ncbi.nlm.nih.gov/geo/query/acc.cgi?acc=GSM503950), [GSM503951](https://www.ncbi.nlm.nih.gov/geo/query/acc.cgi?acc=GSM503951), [GSM503952](https://www.ncbi.nlm.nih.gov/geo/query/acc.cgi?acc=GSM503952), [GSM503953](https://www.ncbi.nlm.nih.gov/geo/query/acc.cgi?acc=GSM503953), [GSM503954](https://www.ncbi.nlm.nih.gov/geo/query/acc.cgi?acc=GSM503954), [GSM503955](https://www.ncbi.nlm.nih.gov/geo/query/acc.cgi?acc=GSM503955), [GSM503956](https://www.ncbi.nlm.nih.gov/geo/query/acc.cgi?acc=GSM503956), [GSM503957](https://www.ncbi.nlm.nih.gov/geo/query/acc.cgi?acc=GSM503957), [GSM503958](https://www.ncbi.nlm.nih.gov/geo/query/acc.cgi?acc=GSM503958), [GSM503959](https://www.ncbi.nlm.nih.gov/geo/query/acc.cgi?acc=GSM503959), [GSM503960](https://www.ncbi.nlm.nih.gov/geo/query/acc.cgi?acc=GSM503960), [GSM503961](https://www.ncbi.nlm.nih.gov/geo/query/acc.cgi?acc=GSM503961), [GSM503962](https://www.ncbi.nlm.nih.gov/geo/query/acc.cgi?acc=GSM5039562), [GSM503963](https://www.ncbi.nlm.nih.gov/geo/query/acc.cgi?acc=GSM503963), [GSM503964](https://www.ncbi.nlm.nih.gov/geo/query/acc.cgi?acc=GSM503964), [GSM503965](https://www.ncbi.nlm.nih.gov/geo/query/acc.cgi?acc=GSM503965), [GSM503966](https://www.ncbi.nlm.nih.gov/geo/query/acc.cgi?acc=GSM503966), [GSM503967](https://www.ncbi.nlm.nih.gov/geo/query/acc.cgi?acc=GSM503967)).

[GSE20164](https://www.ncbi.nlm.nih.gov/geo/query/acc.cgi?acc=GSE20164) (using datasets [GSM506013](https://www.ncbi.nlm.nih.gov/geo/query/acc.cgi?acc=GSM506013), [GSM506014](https://www.ncbi.nlm.nih.gov/geo/query/acc.cgi?acc=GSM506014), [GSM506015](https://www.ncbi.nlm.nih.gov/geo/query/acc.cgi?acc=GSM506015), [GSM506016](https://www.ncbi.nlm.nih.gov/geo/query/acc.cgi?acc=GSM506016), [GSM506017](https://www.ncbi.nlm.nih.gov/geo/query/acc.cgi?acc=GSM506017), [GSM506018](https://www.ncbi.nlm.nih.gov/geo/query/acc.cgi?acc=GSM506018), [GSM506019](https://www.ncbi.nlm.nih.gov/geo/query/acc.cgi?acc=GSM506019), [GSM506020](https://www.ncbi.nlm.nih.gov/geo/query/acc.cgi?acc=GSM506020), [GSM506021](https://www.ncbi.nlm.nih.gov/geo/query/acc.cgi?acc=GSM506021), [GSM506022)](https://www.ncbi.nlm.nih.gov/geo/query/acc.cgi?acc=GSM506022).

[GSE20333](https://www.ncbi.nlm.nih.gov/geo/query/acc.cgi?acc=GSE20333) (using datasets [GSM509547](https://www.ncbi.nlm.nih.gov/geo/query/acc.cgi?acc=GSM509547), [GSM509548](https://www.ncbi.nlm.nih.gov/geo/query/acc.cgi?acc=GSM509548), [GSM509549](https://www.ncbi.nlm.nih.gov/geo/query/acc.cgi?acc=GSM509549), [GSM509550](https://www.ncbi.nlm.nih.gov/geo/query/acc.cgi?acc=GSM509550), [GSM509551](https://www.ncbi.nlm.nih.gov/geo/query/acc.cgi?acc=GSM509551), [GSM509552](https://www.ncbi.nlm.nih.gov/geo/query/acc.cgi?acc=GSM509552), [GSM509553](https://www.ncbi.nlm.nih.gov/geo/query/acc.cgi?acc=GSM509553), [GSM509554](https://www.ncbi.nlm.nih.gov/geo/query/acc.cgi?acc=GSM509554), [GSM509555](https://www.ncbi.nlm.nih.gov/geo/query/acc.cgi?acc=GSM509555), [GSM509556](https://www.ncbi.nlm.nih.gov/geo/query/acc.cgi?acc=GSM509556), [GSM509557](https://www.ncbi.nlm.nih.gov/geo/query/acc.cgi?acc=GSM509557), [GSM509558](https://www.ncbi.nlm.nih.gov/geo/query/acc.cgi?acc=GSM509558))

[GSE7307](https://www.ncbi.nlm.nih.gov/geo/query/acc.cgi?acc=GSE7307) (using datasets [GSM506013](https://www.ncbi.nlm.nih.gov/geo/query/acc.cgi?acc=GSM506013), [GSM506014](https://www.ncbi.nlm.nih.gov/geo/query/acc.cgi?acc=GSM506014), [GSM506015](https://www.ncbi.nlm.nih.gov/geo/query/acc.cgi?acc=GSM506015), [GSM506016](https://www.ncbi.nlm.nih.gov/geo/query/acc.cgi?acc=GSM506016), [GSM506017](https://www.ncbi.nlm.nih.gov/geo/query/acc.cgi?acc=GSM506017), [GSM506018](https://www.ncbi.nlm.nih.gov/geo/query/acc.cgi?acc=GSM506018), [GSM506019](https://www.ncbi.nlm.nih.gov/geo/query/acc.cgi?acc=GSM506019), [GSM506020](https://www.ncbi.nlm.nih.gov/geo/query/acc.cgi?acc=GSM506020), [GSM506021](https://www.ncbi.nlm.nih.gov/geo/query/acc.cgi?acc=GSM506021), [GSM506022)](https://www.ncbi.nlm.nih.gov/geo/query/acc.cgi?acc=GSM506022).

[GSE7621](https://www.ncbi.nlm.nih.gov/geo/query/acc.cgi?acc=GSE7621) (using datasets [GSM184354](https://www.ncbi.nlm.nih.gov/geo/query/acc.cgi?acc=GSM184354), [GSM184355](https://www.ncbi.nlm.nih.gov/geo/query/acc.cgi?acc=GSM184355), [GSM184356](https://www.ncbi.nlm.nih.gov/geo/query/acc.cgi?acc=GSM184356), [GSM184357](https://www.ncbi.nlm.nih.gov/geo/query/acc.cgi?acc=GSM184357), [GSM184358](https://www.ncbi.nlm.nih.gov/geo/query/acc.cgi?acc=GSM184358), [GSM184359](https://www.ncbi.nlm.nih.gov/geo/query/acc.cgi?acc=GSM184359), [GSM184360](https://www.ncbi.nlm.nih.gov/geo/query/acc.cgi?acc=GSM184360), [GSM184361](https://www.ncbi.nlm.nih.gov/geo/query/acc.cgi?acc=GSM184361), [GSM184362](https://www.ncbi.nlm.nih.gov/geo/query/acc.cgi?acc=GSM184362), [GSM184363](https://www.ncbi.nlm.nih.gov/geo/query/acc.cgi?acc=GSM184363), [GSM184364](https://www.ncbi.nlm.nih.gov/geo/query/acc.cgi?acc=GSM184364), [GSM184365](https://www.ncbi.nlm.nih.gov/geo/query/acc.cgi?acc=GSM184365), [GSM184366](https://www.ncbi.nlm.nih.gov/geo/query/acc.cgi?acc=GSM184366), [GSM184367](https://www.ncbi.nlm.nih.gov/geo/query/acc.cgi?acc=GSM184367), [GSM184368](https://www.ncbi.nlm.nih.gov/geo/query/acc.cgi?acc=GSM184368), [GSM184369](https://www.ncbi.nlm.nih.gov/geo/query/acc.cgi?acc=GSM184369), [GSM184370](https://www.ncbi.nlm.nih.gov/geo/query/acc.cgi?acc=GSM184370), [GSM184371](https://www.ncbi.nlm.nih.gov/geo/query/acc.cgi?acc=GSM184371), [GSM184372](https://www.ncbi.nlm.nih.gov/geo/query/acc.cgi?acc=GSM184372), [GSM184373](https://www.ncbi.nlm.nih.gov/geo/query/acc.cgi?acc=GSM184373), [GSM184374](https://www.ncbi.nlm.nih.gov/geo/query/acc.cgi?acc=GSM184374), [GSM184375](https://www.ncbi.nlm.nih.gov/geo/query/acc.cgi?acc=GSM184375), [GSM184376](https://www.ncbi.nlm.nih.gov/geo/query/acc.cgi?acc=GSM184376), [GSM184377](https://www.ncbi.nlm.nih.gov/geo/query/acc.cgi?acc=GSM184377), [GSM184378](https://www.ncbi.nlm.nih.gov/geo/query/acc.cgi?acc=GSM184378)).

[GDS3128](https://www.ncbi.nlm.nih.gov/geo/query/acc.cgi?acc=GDS3128) (using datasets [GSM208630](https://www.ncbi.nlm.nih.gov/geo/query/acc.cgi?acc=GSM208630), [GSM208631](https://www.ncbi.nlm.nih.gov/geo/query/acc.cgi?acc=GSM208631), [GSM208632](https://www.ncbi.nlm.nih.gov/geo/query/acc.cgi?acc=GSM208632), [GSM208633](https://www.ncbi.nlm.nih.gov/geo/query/acc.cgi?acc=GSM208633), [GSM208634](https://www.ncbi.nlm.nih.gov/geo/query/acc.cgi?acc=GSM208634), [GSM208635](https://www.ncbi.nlm.nih.gov/geo/query/acc.cgi?acc=GSM208635), [GSM208645](https://www.ncbi.nlm.nih.gov/geo/query/acc.cgi?acc=GSM208645), [GSM208646](https://www.ncbi.nlm.nih.gov/geo/query/acc.cgi?acc=GSM208646), [GSM208647](https://www.ncbi.nlm.nih.gov/geo/query/acc.cgi?acc=GSM208647), [GSM208648](https://www.ncbi.nlm.nih.gov/geo/query/acc.cgi?acc=GSM208648), [GSM208649](https://www.ncbi.nlm.nih.gov/geo/query/acc.cgi?acc=GSM208649), [GSM208650](https://www.ncbi.nlm.nih.gov/geo/query/acc.cgi?acc=GSM208650), [GSM208651](https://www.ncbi.nlm.nih.gov/geo/query/acc.cgi?acc=GSM208651), [GSM208652](https://www.ncbi.nlm.nih.gov/geo/query/acc.cgi?acc=GSM208652), [GSM208668](https://www.ncbi.nlm.nih.gov/geo/query/acc.cgi?acc=GSM208668), [GSM208636](https://www.ncbi.nlm.nih.gov/geo/query/acc.cgi?acc=GSM208636), [GSM208637](https://www.ncbi.nlm.nih.gov/geo/query/acc.cgi?acc=GSM208637), [GSM208638](https://www.ncbi.nlm.nih.gov/geo/query/acc.cgi?acc=GSM208638), [GSM208639](https://www.ncbi.nlm.nih.gov/geo/query/acc.cgi?acc=GSM208639), [GSM208640](https://www.ncbi.nlm.nih.gov/geo/query/acc.cgi?acc=GSM208640), [GSM208641](https://www.ncbi.nlm.nih.gov/geo/query/acc.cgi?acc=GSM208641), [GSM208642](https://www.ncbi.nlm.nih.gov/geo/query/acc.cgi?acc=GSM208642), [GSM208643](https://www.ncbi.nlm.nih.gov/geo/query/acc.cgi?acc=GSM208643), [GSM208644](https://www.ncbi.nlm.nih.gov/geo/query/acc.cgi?acc=GSM208644), [GSM208653](https://www.ncbi.nlm.nih.gov/geo/query/acc.cgi?acc=GSM208653), [GSM208654](https://www.ncbi.nlm.nih.gov/geo/query/acc.cgi?acc=GSM208654), [GSM208655](https://www.ncbi.nlm.nih.gov/geo/query/acc.cgi?acc=GSM208655), [GSM208656](https://www.ncbi.nlm.nih.gov/geo/query/acc.cgi?acc=GSM208656), [GSM208657](https://www.ncbi.nlm.nih.gov/geo/query/acc.cgi?acc=GSM208657), [GSM208658](https://www.ncbi.nlm.nih.gov/geo/query/acc.cgi?acc=GSM208658), [GSM208659](https://www.ncbi.nlm.nih.gov/geo/query/acc.cgi?acc=GSM208659), [GSM208660](https://www.ncbi.nlm.nih.gov/geo/query/acc.cgi?acc=GSM208660), [GSM208661](https://www.ncbi.nlm.nih.gov/geo/query/acc.cgi?acc=GSM208661), [GSM208662](https://www.ncbi.nlm.nih.gov/geo/query/acc.cgi?acc=GSM208662), [GSM208663](https://www.ncbi.nlm.nih.gov/geo/query/acc.cgi?acc=GSM208663), [GSM208664](https://www.ncbi.nlm.nih.gov/geo/query/acc.cgi?acc=GSM208664), [GSM208665](https://www.ncbi.nlm.nih.gov/geo/query/acc.cgi?acc=GSM208665), [GSM208666](https://www.ncbi.nlm.nih.gov/geo/query/acc.cgi?acc=GSM208666), [GSM208667](https://www.ncbi.nlm.nih.gov/geo/query/acc.cgi?acc=GSM208667)).

[GDS3129](https://www.ncbi.nlm.nih.gov/geo/query/acc.cgi?acc=GDS3128) (this study used the same samples as [GDS3128](https://www.ncbi.nlm.nih.gov/geo/query/acc.cgi?acc=GDS3128) but using a different platform GPL97): in this study we only used GPL97 data when GPL96 data was unavailable).

[GSE54282](https://www.ncbi.nlm.nih.gov/geo/query/acc.cgi?acc=GSE54282) (using datasets [GSM1311807](https://www.ncbi.nlm.nih.gov/geo/query/acc.cgi?acc=GSM1311807), [GSM1311810](https://www.ncbi.nlm.nih.gov/geo/query/acc.cgi?acc=GSM1311810), [GSM1311813](https://www.ncbi.nlm.nih.gov/geo/query/acc.cgi?acc=GSM1311813), [GSM1311815](https://www.ncbi.nlm.nih.gov/geo/query/acc.cgi?acc=GSM1311815), [GSM1311818](https://www.ncbi.nlm.nih.gov/geo/query/acc.cgi?acc=GSM1311818), [GSM1311821](https://www.ncbi.nlm.nih.gov/geo/query/acc.cgi?acc=GSM1311821)).

[GSE43490](https://www.ncbi.nlm.nih.gov/geo/query/acc.cgi?acc=GSE43490) (using datasets [GSM1294118](https://www.ncbi.nlm.nih.gov/geo/query/acc.cgi?acc=GSM1294118), [GSM1294119](https://www.ncbi.nlm.nih.gov/geo/query/acc.cgi?acc=GSM1294119), [GSM1294120](https://www.ncbi.nlm.nih.gov/geo/query/acc.cgi?acc=GSM1294120), [GSM1294121](https://www.ncbi.nlm.nih.gov/geo/query/acc.cgi?acc=GSM1294121), [GSM1294122](https://www.ncbi.nlm.nih.gov/geo/query/acc.cgi?acc=GSM1294122), [GSM1294123](https://www.ncbi.nlm.nih.gov/geo/query/acc.cgi?acc=GSM1294123), [GSM1294124](https://www.ncbi.nlm.nih.gov/geo/query/acc.cgi?acc=GSM1294124), [GSM1294125](https://www.ncbi.nlm.nih.gov/geo/query/acc.cgi?acc=GSM1294125), [GSM1294126](https://www.ncbi.nlm.nih.gov/geo/query/acc.cgi?acc=GSM1294126), [GSM1294127](https://www.ncbi.nlm.nih.gov/geo/query/acc.cgi?acc=GSM1294127), [GSM1294128](https://www.ncbi.nlm.nih.gov/geo/query/acc.cgi?acc=GSM1294128), [GSM1294129](https://www.ncbi.nlm.nih.gov/geo/query/acc.cgi?acc=GSM1294129), [GSM1294130](https://www.ncbi.nlm.nih.gov/geo/query/acc.cgi?acc=GSM1294130)).

[GSE8397](https://www.ncbi.nlm.nih.gov/geo/query/acc.cgi?acc=GSE8397) (using datasets [GSM208630](https://www.ncbi.nlm.nih.gov/geo/query/acc.cgi?acc=GSM208630), [GSM208631](https://www.ncbi.nlm.nih.gov/geo/query/acc.cgi?acc=GSM208631), [GSM208632](https://www.ncbi.nlm.nih.gov/geo/query/acc.cgi?acc=GSM208632), [GSM208633](https://www.ncbi.nlm.nih.gov/geo/query/acc.cgi?acc=GSM208633), [GSM208634](https://www.ncbi.nlm.nih.gov/geo/query/acc.cgi?acc=GSM208634), [GSM208635](https://www.ncbi.nlm.nih.gov/geo/query/acc.cgi?acc=GSM208635), [GSM208636](https://www.ncbi.nlm.nih.gov/geo/query/acc.cgi?acc=GSM208636), [GSM208637](https://www.ncbi.nlm.nih.gov/geo/query/acc.cgi?acc=GSM208637), [GSM208638](https://www.ncbi.nlm.nih.gov/geo/query/acc.cgi?acc=GSM208638), [GSM208639](https://www.ncbi.nlm.nih.gov/geo/query/acc.cgi?acc=GSM208639), [GSM208640](https://www.ncbi.nlm.nih.gov/geo/query/acc.cgi?acc=GSM208640), [GSM208641](https://www.ncbi.nlm.nih.gov/geo/query/acc.cgi?acc=GSM208641), [GSM208642](https://www.ncbi.nlm.nih.gov/geo/query/acc.cgi?acc=GSM208642), [GSM208643](https://www.ncbi.nlm.nih.gov/geo/query/acc.cgi?acc=GSM208643), [GSM208644](https://www.ncbi.nlm.nih.gov/geo/query/acc.cgi?acc=GSM208644), [GSM208645](https://www.ncbi.nlm.nih.gov/geo/query/acc.cgi?acc=GSM208645), [GSM208646](https://www.ncbi.nlm.nih.gov/geo/query/acc.cgi?acc=GSM208646), [GSM208647](https://www.ncbi.nlm.nih.gov/geo/query/acc.cgi?acc=GSM208647), [GSM208648](https://www.ncbi.nlm.nih.gov/geo/query/acc.cgi?acc=GSM208648), [GSM208649](https://www.ncbi.nlm.nih.gov/geo/query/acc.cgi?acc=GSM208649), [GSM208650](https://www.ncbi.nlm.nih.gov/geo/query/acc.cgi?acc=GSM208650), [GSM208651](https://www.ncbi.nlm.nih.gov/geo/query/acc.cgi?acc=GSM208651), [GSM208652](https://www.ncbi.nlm.nih.gov/geo/query/acc.cgi?acc=GSM208652), [GSM208653](https://www.ncbi.nlm.nih.gov/geo/query/acc.cgi?acc=GSM208653), [GSM208654](https://www.ncbi.nlm.nih.gov/geo/query/acc.cgi?acc=GSM208654), [GSM208655](https://www.ncbi.nlm.nih.gov/geo/query/acc.cgi?acc=GSM208655), [GSM208656](https://www.ncbi.nlm.nih.gov/geo/query/acc.cgi?acc=GSM208656), [GSM208657](https://www.ncbi.nlm.nih.gov/geo/query/acc.cgi?acc=GSM208657), [GSM208658](https://www.ncbi.nlm.nih.gov/geo/query/acc.cgi?acc=GSM208658), [GSM208659](https://www.ncbi.nlm.nih.gov/geo/query/acc.cgi?acc=GSM208659), [GSM208660](https://www.ncbi.nlm.nih.gov/geo/query/acc.cgi?acc=GSM208660), [GSM208661](https://www.ncbi.nlm.nih.gov/geo/query/acc.cgi?acc=GSM208661), [GSM208662](https://www.ncbi.nlm.nih.gov/geo/query/acc.cgi?acc=GSM208662), [GSM208663](https://www.ncbi.nlm.nih.gov/geo/query/acc.cgi?acc=GSM208663), [GSM208664](https://www.ncbi.nlm.nih.gov/geo/query/acc.cgi?acc=GSM208664), [GSM208665](https://www.ncbi.nlm.nih.gov/geo/query/acc.cgi?acc=GSM208665), [GSM208666](https://www.ncbi.nlm.nih.gov/geo/query/acc.cgi?acc=GSM208666), [GSM208667](https://www.ncbi.nlm.nih.gov/geo/query/acc.cgi?acc=GSM208667), [GSM208668](https://www.ncbi.nlm.nih.gov/geo/query/acc.cgi?acc=GSM208668), [GSM208677](https://www.ncbi.nlm.nih.gov/geo/query/acc.cgi?acc=GSM208677), [GSM208678](https://www.ncbi.nlm.nih.gov/geo/query/acc.cgi?acc=GSM208678), [GSM208679](https://www.ncbi.nlm.nih.gov/geo/query/acc.cgi?acc=GSM208679), [GSM208680](https://www.ncbi.nlm.nih.gov/geo/query/acc.cgi?acc=GSM208680), [GSM208681](https://www.ncbi.nlm.nih.gov/geo/query/acc.cgi?acc=GSM208681), [GSM208682](https://www.ncbi.nlm.nih.gov/geo/query/acc.cgi?acc=GSM208682), [GSM208683](https://www.ncbi.nlm.nih.gov/geo/query/acc.cgi?acc=GSM208683), [GSM208684](https://www.ncbi.nlm.nih.gov/geo/query/acc.cgi?acc=GSM208684), [GSM208685](https://www.ncbi.nlm.nih.gov/geo/query/acc.cgi?acc=GSM208685)*,* [GSM208686](https://www.ncbi.nlm.nih.gov/geo/query/acc.cgi?acc=GSM208686)*,* [GSM208687](https://www.ncbi.nlm.nih.gov/geo/query/acc.cgi?acc=GSM208687), [GSM208688](https://www.ncbi.nlm.nih.gov/geo/query/acc.cgi?acc=GSM208688), [GSM208689](https://www.ncbi.nlm.nih.gov/geo/query/acc.cgi?acc=GSM208689), [GSM208690](https://www.ncbi.nlm.nih.gov/geo/query/acc.cgi?acc=GSM208690), [GSM208691](https://www.ncbi.nlm.nih.gov/geo/query/acc.cgi?acc=GSM208691), [GSM208692](https://www.ncbi.nlm.nih.gov/geo/query/acc.cgi?acc=GSM208692), [GSM208693](https://www.ncbi.nlm.nih.gov/geo/query/acc.cgi?acc=GSM208693), [GSM208694](https://www.ncbi.nlm.nih.gov/geo/query/acc.cgi?acc=GSM208694), [GSM208695](https://www.ncbi.nlm.nih.gov/geo/query/acc.cgi?acc=GSM208695), [GSM208696](https://www.ncbi.nlm.nih.gov/geo/query/acc.cgi?acc=GSM208696), [GSM208697](https://www.ncbi.nlm.nih.gov/geo/query/acc.cgi?acc=GSM208697), [GSM208698](https://www.ncbi.nlm.nih.gov/geo/query/acc.cgi?acc=GSM208698), [GSM208699](https://www.ncbi.nlm.nih.gov/geo/query/acc.cgi?acc=GSM208699), [GSM208700](https://www.ncbi.nlm.nih.gov/geo/query/acc.cgi?acc=GSM208700), [GSM208701](https://www.ncbi.nlm.nih.gov/geo/query/acc.cgi?acc=GSM208701), ,[GSM208702](https://www.ncbi.nlm.nih.gov/geo/query/acc.cgi?acc=GSM208702), [GSM208703](https://www.ncbi.nlm.nih.gov/geo/query/acc.cgi?acc=GSM208703), [GSM208704](https://www.ncbi.nlm.nih.gov/geo/query/acc.cgi?acc=GSM208704), [GSM208705](https://www.ncbi.nlm.nih.gov/geo/query/acc.cgi?acc=GSM208705), [GSM208706](https://www.ncbi.nlm.nih.gov/geo/query/acc.cgi?acc=GSM208706), [GSM208707](https://www.ncbi.nlm.nih.gov/geo/query/acc.cgi?acc=GSM208707), [GSM208708](https://www.ncbi.nlm.nih.gov/geo/query/acc.cgi?acc=GSM208708), [GSM208709](https://www.ncbi.nlm.nih.gov/geo/query/acc.cgi?acc=GSM208709), [GSM208710](https://www.ncbi.nlm.nih.gov/geo/query/acc.cgi?acc=GSM208710), [GSM208711](https://www.ncbi.nlm.nih.gov/geo/query/acc.cgi?acc=GSM208711), [GSM208712](https://www.ncbi.nlm.nih.gov/geo/query/acc.cgi?acc=GSM208712), [GSM208713](https://www.ncbi.nlm.nih.gov/geo/query/acc.cgi?acc=GSM208713), [GSM208714](https://www.ncbi.nlm.nih.gov/geo/query/acc.cgi?acc=GSM208714), [GSM208715](https://www.ncbi.nlm.nih.gov/geo/query/acc.cgi?acc=GSM208715)).
