# Supplementary material for: Inhibition of β-catenin dependent WNT signalling upregulates the transcriptional repressor NR0B1 and downregulates markers of an A9 phenotype in human embryonic stem cell-derived dopaminergic neurons: Implications for Parkinson’s disease
Source: PLoS One. 2021 Dec 23;16(12):e0261730. doi: 10.1371/journal.pone.0261730 (PMC8700011; doi:10.1371/journal.pone.0261730)
Supplement: S2 Table — (DOCX) [file pone.0261730.s002.docx]

|  | Gene expression ID |
| --- | --- |
| ALDH1A1 | Hs00946916_m1 |
| BDNF | Hs03805856_g1 |
| CALB1 | Hs01077197_m1 |
| DKK3 | Hs00951304_m1 |
| EN1 | Hs00154977_m1 |
| EN2 | Hs00171321_m1 |
| FOXA2 | Hs00232764_m1 |
| GDNF | Hs01055329_m1 |
| GFAP | Hs03805856_g1 |
| HPRT1 | Hs02800695_m1 |
| KCJN6 | Hs01040524_m1 |
| LMX1A | Hs00892663_m1 |
| MAP2 | Hs00258900_m1 |
| MSX1 | Hs00427183_m1 |
| MSX2 | Hs00751239_s1 |
| NES | Hs04187831_g1 |
| NEUROD1 | Hs01922995_s1 |
| NR0B1 | Hs03043658_m1 |
| NR0B2 | Hs00222677_m1 |
| NR4A2 | Hs01118813_m1 |
| OTX2 | Hs00222238_m1 |
| PAX6 | Hs01088114_m1 |
| PITX3 | Hs00374504_m1 |
| S100B | Hs00902901_m1 |
| SLC18A2 | Hs00996834_m1 |
| SLC6A3 | Hs00997364_m1 |
| SNCA | Hs00240906_m1 |
| SOX6 | Hs00264525_m1 |
| TBP1 | Hs00427620_m1 |
| TH | Hs00165941_m1 |
| TUBB3 | Hs00801390_s1 |
| WNT1 | Hs01011247_m1 |
| WNT5A | Hs00998537_m1 |
