# Supplementary material for: Inhibition of β-catenin dependent WNT signalling upregulates the transcriptional repressor NR0B1 and downregulates markers of an A9 phenotype in human embryonic stem cell-derived dopaminergic neurons: Implications for Parkinson’s disease
Source: PLoS One. 2021 Dec 23;16(12):e0261730. doi: 10.1371/journal.pone.0261730 (PMC8700011; doi:10.1371/journal.pone.0261730)
Supplement: S4 Table — (DOCX) [file pone.0261730.s004.docx]

| Antibody | Supplier | Catalogue number |
| --- | --- | --- |
| Anti-beta III Tubulin (chicken) | Abcam | ab41489 |
| Anti-Tyrosine Hydroxylase (sheep) | Abcam | ab113 |
| Anti-Map2 (chicken) | Abcam | ab5392 |
| Anti-NeuroD1 (rabbit) | Abcam | ab236148 |
| Anti-NR0B1 (rabbit) | Thermofisher | PA5-80865 |
| Anti-NR0B2 (rabbit) | Thermofisher | PA5-102494 |
| Anti-PITX3 (rabbit) | ThermoFisher | 38-2850 |
| Anti-KCNJ6 (rabbit) | ThermoFisher | PA5-77575 |
| Anti-SLC6A3 (rabbit) | ThermoFisher | PA5-106751 |
|  |  |  |
| Donkey Anti-Rabbit IgG H&L (Alexa Fluor® 488) | Abcam | ab150073 |
| Donkey Anti Sheep IgG HL Alexa Fluor 555 | Abcam | ab150178 |
| Donkey Anti-Rabbit IgG H&L (Alexa Fluor® 555) | Abcam | Ab150062 |
| Donkey Anti-Chicken IgY 647 | Jackson | 703-605-155 |
