# Supplementary figures and images for: Inhibition of β-catenin dependent WNT signalling upregulates the transcriptional repressor NR0B1 and downregulates markers of an A9 phenotype in human embryonic stem cell-derived dopaminergic neurons: Implications for Parkinson’s disease
Source: PLoS One. 2021 Dec 23;16(12):e0261730. doi: 10.1371/journal.pone.0261730 (PMC8700011; doi:10.1371/journal.pone.0261730)

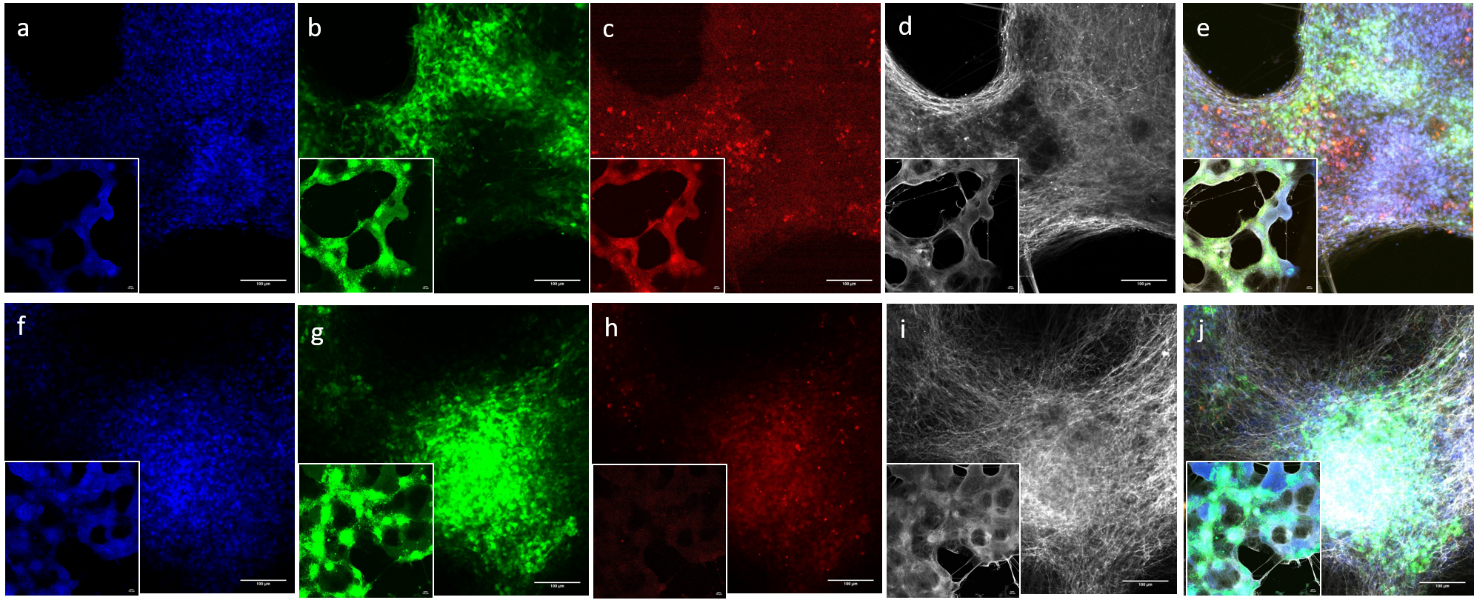

Supplement: S1 Fig — Panels (a)-(e) show Hoechst nuclear labelling, LMX1A-eGFP, NEUROD1, β3-tubulin and colour combined images seven days after NEUROD1 transfection. Panels (f)-(j) show Hoechst nuclear labelling, LMX1A-eGFP, NEUROD1, β3-tubulin and colour combined images seven days after control transfection. Images are all 20x (scale bar is 100μm) taken with the same imaging parameters. The insets show x4 images. (TIF) [file pone.0261730.s006.tif]

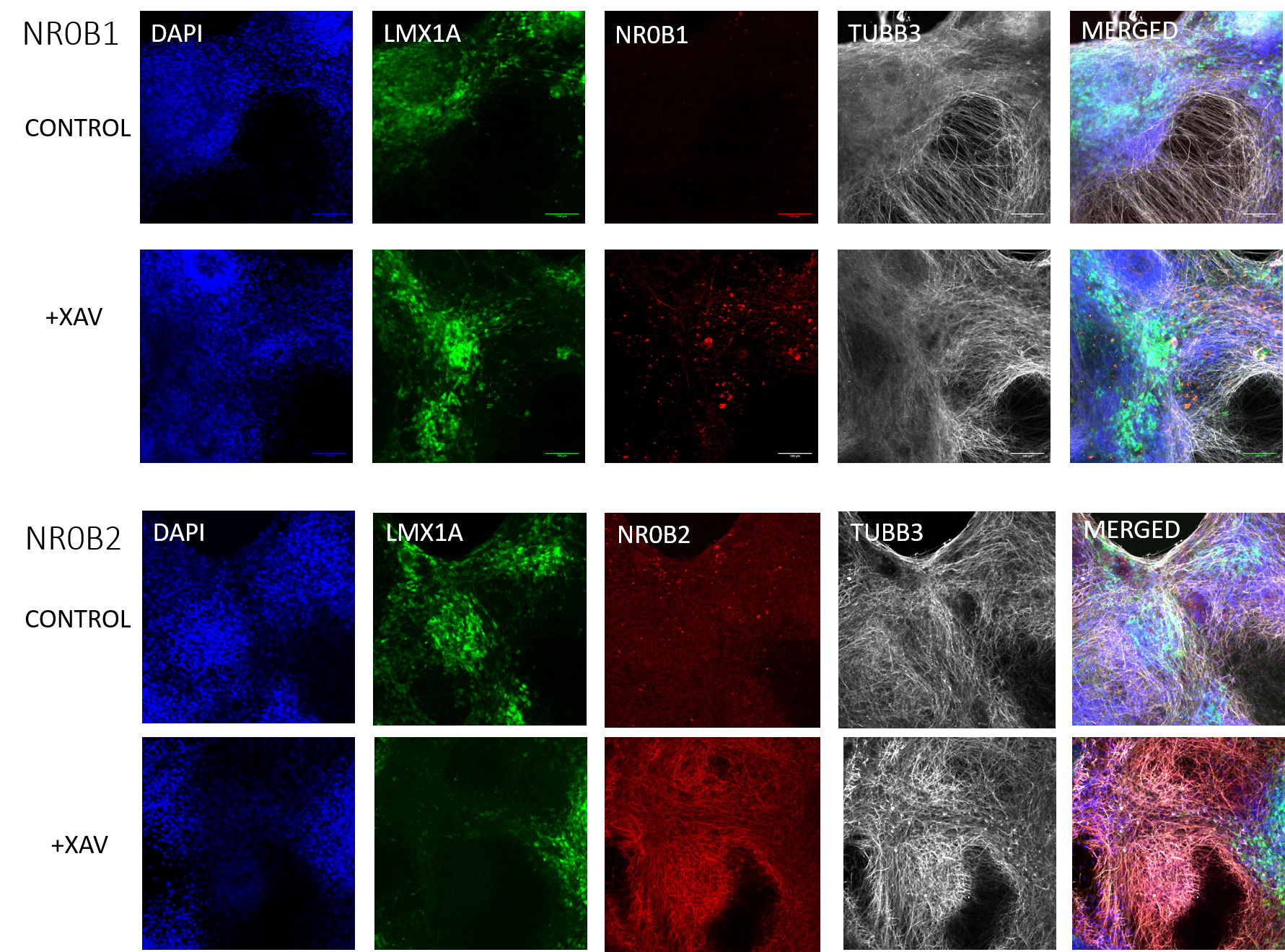

Supplement: S2 Fig — From left to right the top pair of panels show DAPI nuclear labelling, LMX1A-eGFP, NR0B1, β3-tubulin and colour combined images after 14 days of vehicle (control, upper) or XAV (100nM, lower). The bottom pair of panels show identical labelling except where NR0B2 is indicated. Images are all 20x (scale bar is 100μm) taken with the same imaging parameters. (TIF) [file pone.0261730.s007.tif]

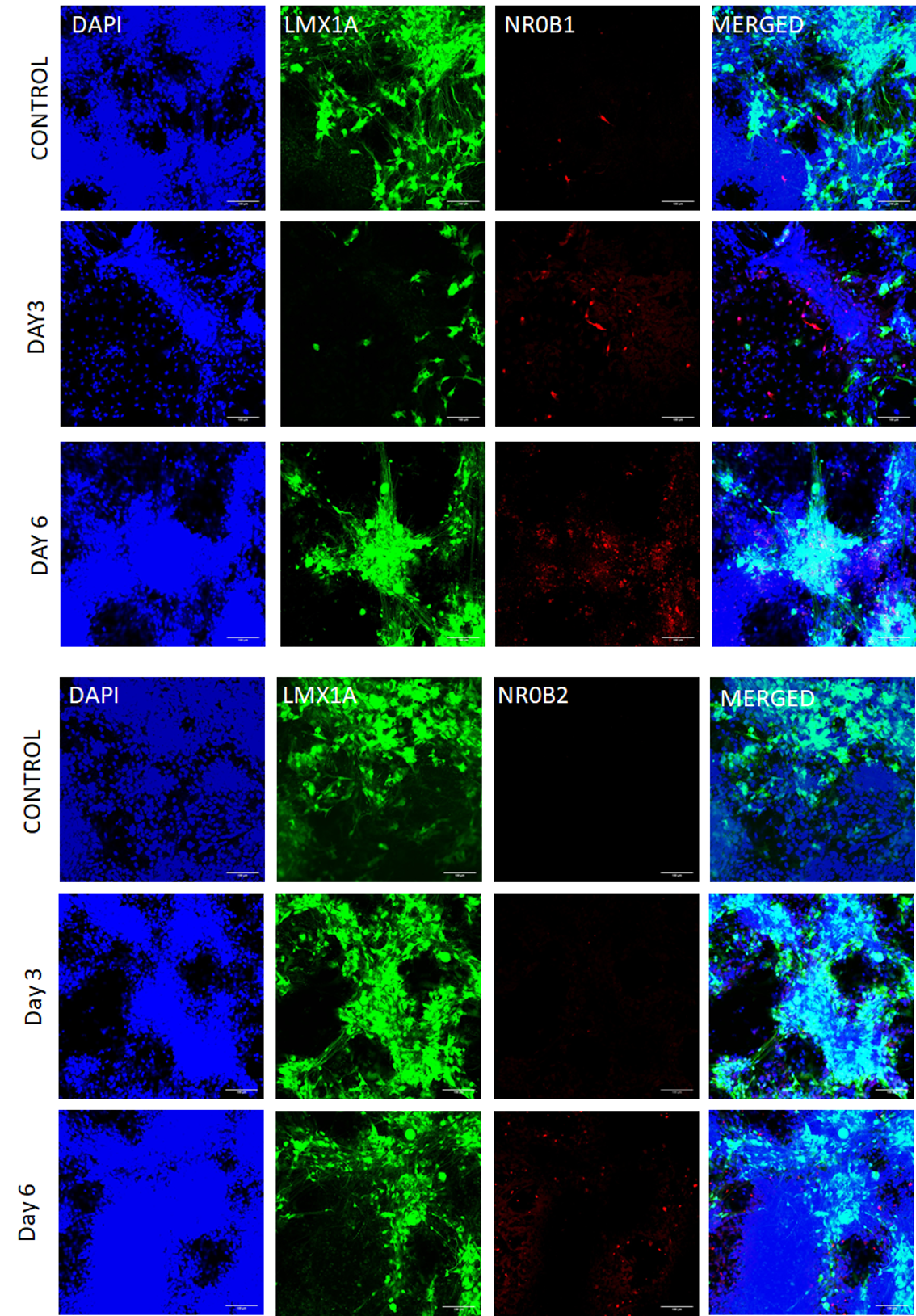

Supplement: S3 Fig — From left to right the panels DAPI nuclear labelling, LMX1A-eGFP, NR0B1 (top three panels) & NR0B2 (bottom three panels) and colour combined images in vehicle (control, 6 days) and at 3 and 6 days after transfection. Images are all 20x (scale bar is 100μm) taken with the same imaging parameters across each of the days. (TIF) [file pone.0261730.s008.tif]

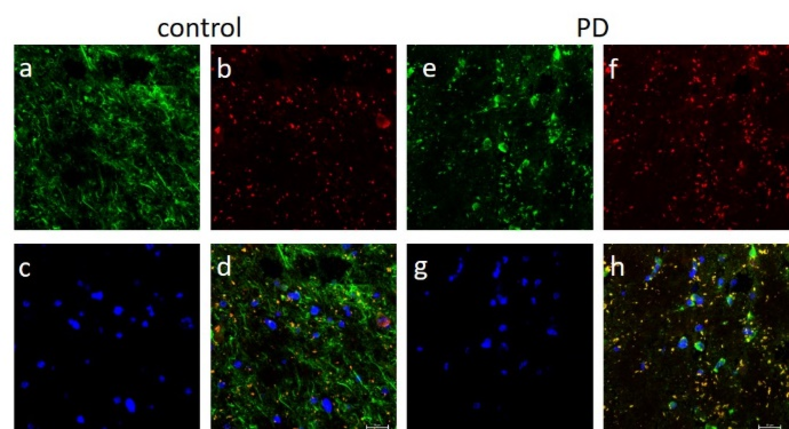

Supplement: S4 Fig — Panels show tyrosine hydroxylase (green, panels a and e) and PITX3 (red, panels b and f) in sections of substantia nigra from an age-matched control (panels a-d) and a PD patient (panels e-h). Panels (c) and (g) show Hoechst labelled nuclei, while panels (d) and (h) show colour combined images. Note the widespread reduction in green fluorescence in PD. Scale bar indicates 25μm. (TIF) [file pone.0261730.s009.tif]

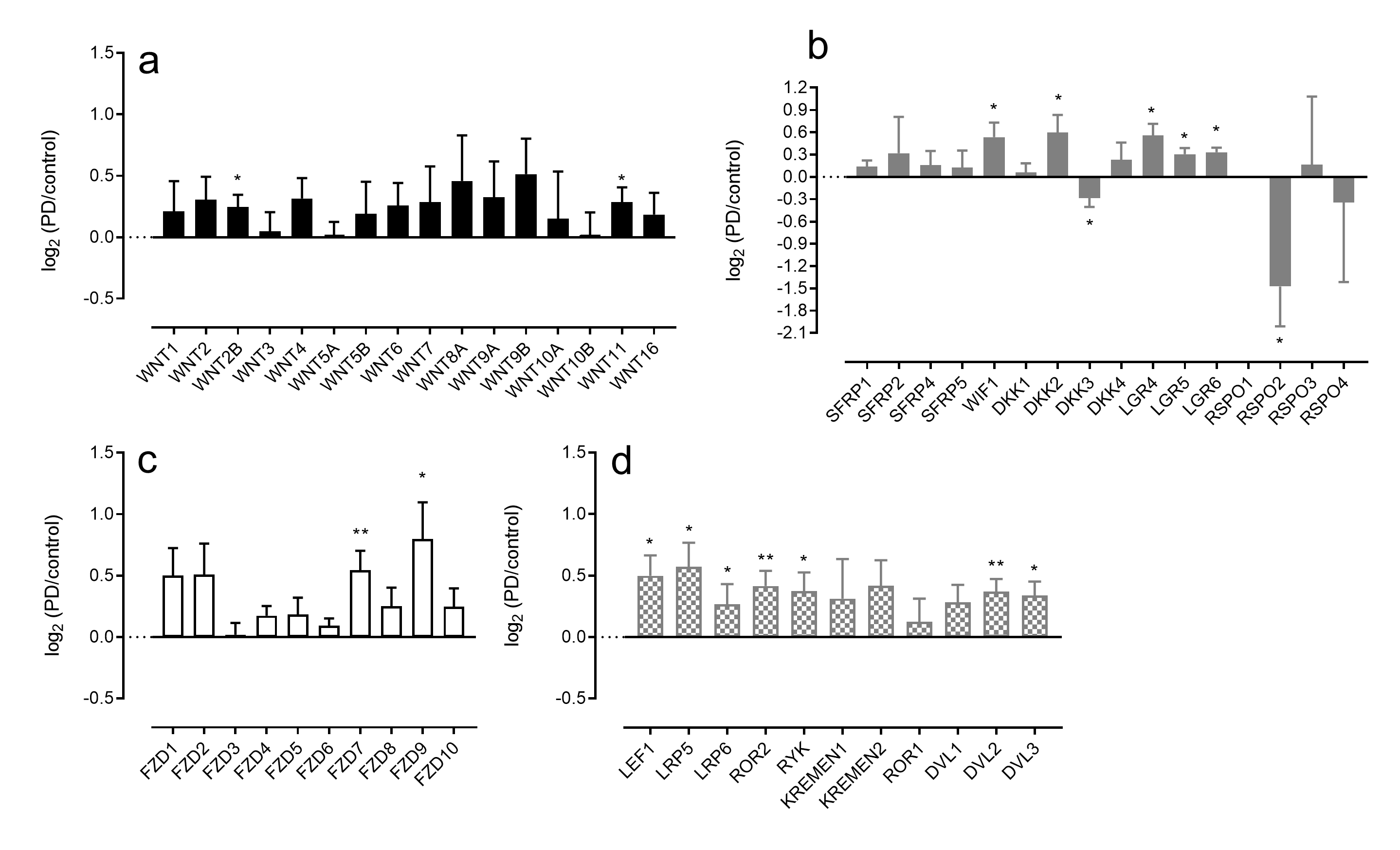

Supplement: S5 Fig — Panel (a) shows changes in nigral WNTs, panel (b) shows changes in soluble WNT signalling ligands while panels (c) and (d) show changes in frizzled receptors and cellular WNT-regulators or signal transduction components. Although data is shown as a ratio PD/control, the ΔCt values for matched control and PD transcript arrays analysed using Student’s paired t-tests. *, ** = P<0.05, 0.01, respectively (n = 6–10 datasets). (TIF) [file pone.0261730.s010.tif]
